# Thwarting Isomerization through Rigidity: A Promising HBED Derivative for the Chelation of Gallium-68

**DOI:** 10.1021/acs.inorgchem.5c00930

**Published:** 2025-07-17

**Authors:** Marianna Tosato, Matteo Boniburini, Francesco Faglioni, Francesco Genua, Matteo Mari, Jennifer Storchi, Sara Franchi, Mattia Asti, Erika Ferrari

**Affiliations:** † Radiopharmaceutical Chemistry Laboratory, Nuclear Medicine Unit, AUSL-IRCCS Reggio Emilia, 42122 Reggio Emilia, Italy; ‡ Department of Chemical and Geological Sciences, University of Modena and Reggio Emilia, 41125 Modena, Italy; § Department of Chemical Sciences, University of Padova, 35131 Padova, Italy

## Abstract

The hexadentate acyclic ligand, N,N′-di­(2-hydroxybenzyl)-(1,2-cyclohexanediamine)-N,N′-diacetic acid (HBCD) designed for the chelation of the positron-emitting radiometal ^68^Ga was developed by replacing the flexible ethylenediamine backbone of its parent ligand, *N*,*N*′-di­(2-hydroxybenzyl)­ethylenediamine-*N*,*N*′-diacetic acid (HBED), with a more rigid cyclohexane diamine scaffold (DACH). This aims to hinder the formation of multiple isomers upon Ga^3+^-complexation as observed in HBED-containing molecules, which could affect the in vivo behavior of ^68^Ga-labeled radiopharmaceuticals. To this end, we report the synthesis of HBCD, a comprehensive investigation of its acid–base behavior, its Ga^3+^ coordination chemistry, its labeling performances with generator-produced ^68^Ga, and the stability of the corresponding radioactive complex in physiological media. Our findings confirm that the DACH scaffold promotes the formation of a hexacoordinated single-isomer Ga^3+^ complex. Although Ga^3+^-HBCD resulted less thermodynamically stable than Ga^3+^-HBED, it is by far more stable than the Ga^3+^ complex formed with the clinical workhorse DOTA chelator. HBCD demonstrated the ability to bind [^68^Ga]­Ga^3+^ under extremely diluted radiochemical conditions (*C*_L_ = 10^–6^ M, 90 °C, pH 4.5 and 7). Notably, [^68^Ga]­[Ga­(HBCD)]^−^ shows exceptional stability in biological media. These results position HBCD as a highly attractive chelator for the development of next-generation PET radiotracers, effectively addressing the issue of isomerization in its parent ligand HBED.

## Graphical abstract

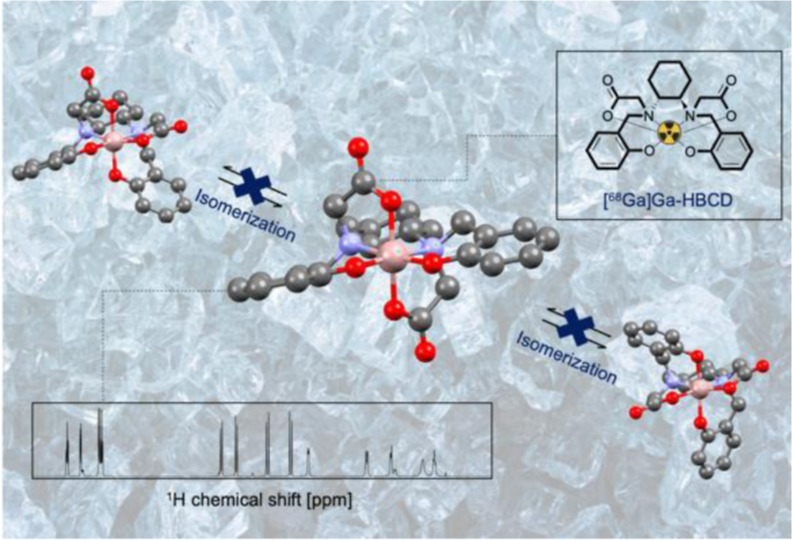

## 1 Introduction

The urgency to prevent, diagnose, and treat cancer has reached a critical point as this disease remains one of the leading causes of death worldwide. Early detection is essential for effective treatment, driving significant efforts in developing innovative imaging technologies and diagnostic agents.^1^ In this context, Positron Emission Tomography (PET) stands out as a ground-breaking technique employing positron-emitting (β^+^) radionuclides to noninvasively visualize functional processes within the body and identify tumor lesions.^2–4^ The growth of preclinical and clinical research and the increasing adoption of PET for early cancer diagnosis and prognosis have catalyzed interest in β^+^ emitters. However, most PET radioisotopes (e.g., fluorine-18) are produced in cyclotrons. This hampers their availability in hospitals that do not have access to such technology bounding them to purchase the radiotracers from external distributors and so hindering the widespread adoption of PET imaging modality.^5^ A notable exception is gallium-68 (^68^Ga), which boasts advantageous decay properties (*I*_β_^+^ = 87.72%, *E*_average_ = 836.0 keV; *I*_β_^+^ = 1.19%, *E*_average_ = 352.6 keV) and convenient half-life (*t*_1/2_ = 67.71 min), along with the availability of commercial germanium-68 (^68^Ge)/^68^Ga generators which can be stored on-site and used in clinical facilities.^6–8^ These properties have allowed to overcome the production-related limitations of other PET radioisotopes, significantly boosting interest in this radiometal.^2–4,9–13^

To harness the properties of ^68^Ga in molecular imaging, it is crucial to efficiently incorporate it into tumor-targeting molecules using a chelating agent. The latter must bind [^68^Ga]­Ga^3+^ forming a radiometal complex with high thermodynamic stability and kinetic inertness to withstand transchelation reaction with biological components (e.g., iron-transporting proteins such as transferrin) or hydrolysis phenomena (e.g., formation of [Ga­(OH)_2_]^+^ at acidic pH or [Ga­(OH)_4_]^−^ under basic conditions), allowing for imaging scans over several hours.^5,9,14,15^ Additionally, the relatively short half-life of ^68^Ga necessitates rapid radiolabeling chemistry to achieve quantitative radiometal incorporation, minimizing the loss of the imaging agent due to decay.^14^

Gallium ions (Ga^3+^) are classified as hard species based on Pearson’s Hard and Soft Acid and Base (HSAB) theory and typically form 6-coordinate complexes.^16^ Thus, hard oxygen donors (O) are ubiquitous in the state-of-the-art [^68^Ga]­Ga^3+^ chelators.

The most widely used ^68^Ga chelator in clinical practice is the macrocycle 1,4,7,10-tetraazacyclododecane-1,4,7,10-tetraacetic acid (DOTA), which, albeit far from being the ideal chelating agent for this radionuclide, has proven its effectiveness in various ^68^Ga-based PET imaging agents.^4,14,15,17–19^ The ubiquity of the [^68^Ga]­Ga-DOTA complex culminated in the approval of two commercial kits for the preparation of ^68^Ga-labeled radiopharmaceuticals for the imaging of GEP-NET (i.e., SomaKit and NetSpot containing DOTATOC and DOTATATE precursor, respectively). However, the incorporation of ^68^Ga with DOTA is a challenging procedure, requiring prolonged incubation (>10 min) at high temperature (*T* = 90 °C).^4,15,19^ These conditions are incompatible with thermosensitive biomolecules and the relatively short half-life of ^68^Ga.^4,5,14,15,19,20^ As a result, many alternative chelators have been explored to overcome these limitations.^15^ For example, the triazacyclononane (TACN)-based chelator 1,4,7-triazacyclononane-1,4,7-triacetic acid (NOTA) represents an important advancement over DOTA, demonstrating quantitative [^68^Ga]­Ga^3+^ binding at room temperature (RT) and acidic environment (pH 3–5.5) within a short time frame (∼10 min).^4,21,22^ Other TACN-derivatives have also been investigated as potential ^68^Ga chelators, such as phosphonate- and phosphinate-containing NOTA analogues.^4,23–25^

Acyclic chelators have also been described for ^68^Ga binding. Examples include 6,6′-{[ethane-1,2-diylbis­(azanediyl)]­bis­(methylene)}­dipicolinic acid (dedpa) and its derivative 6,6′-{[((1*R*,2*R*)-cyclohexane-1,2-diyl)­bis- (azanediyl)]­bis­(methylene)}­dipicolinic acid (CHXdedpa).^26^ While [^68^Ga]­Ga-dedpa exhibits moderate stability in human serum (%[^68^Ga]­Ga-dedpa intact = 77.8% after 2 h), its rigid analogue CHXdedpa demonstrated superior resistance to decomplexation (%[^68^Ga]­Ga-CHXdedpa intact = 90.5% after 2 h).^26^ (6,6′-{[(Carboxymethyl)­azamediyl]­dimethylene}­dipicolinic acid (dpaa) has also been reported to radiolabel [^68^Ga]­Ga^3+^ at neutral pH, but the serum stability of the resulting complex was poor for in vivo imaging.^27,28^ Deferoxamine (DFO) has also been radiolabeled with [^68^Ga]­Ga^3+^, but it is prone to metal dissociation.^4,5^ Among tris­(hydroxypyridinone) chelators, THP^Me^ demonstrated to achieve quantitative radiometal binding in mild conditions (5 min, RT, pH 6.5), resulting in a stable complex^4,14^ that is hitherto under observation as metal *core* for a new generation of radiotracers.^29^

In the plethora of acyclic chelators, *N*,*N*′-di­(2-hydroxybenzyl)­ethylenediamine-*N*,*N*′-diacetic acid (HBED, Figure 1) has emerged as a leading option. First reported by Martell et al. as a chelating ligand for Fe^3+^, HBED can effectively radiolabel [^68^Ga]­Ga^3+^ at room temperature, forming a remarkably stable [^68^Ga]­Ga^3+^ complex.^30–33^

**Figure 1:**
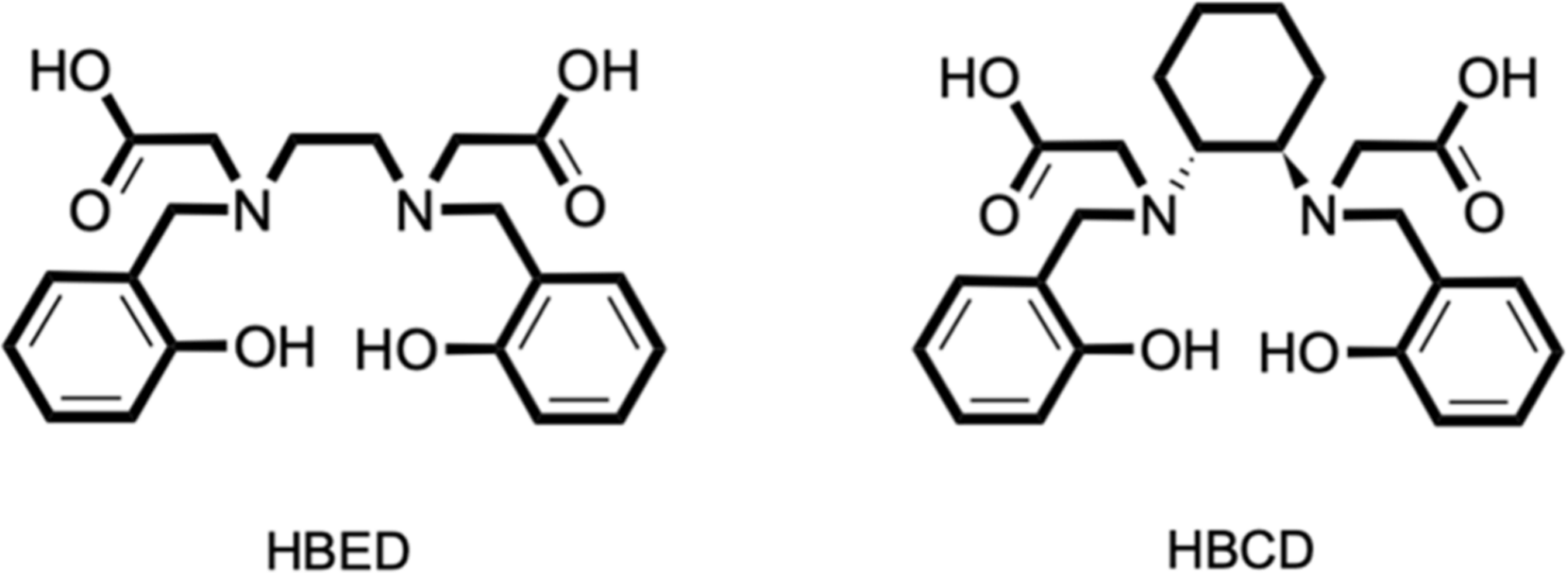
Structure of HBED and HBCD.

The success of HBED and its bifunctional counterpart (*N*,*N*′-bis-[2-hydroxy-5-(carboxyethyl)­benzyl]­ethylenediamine-*N*,*N*′-diacetic acid, HBED-CC) have culminated in 2020 in the FDA approval of [^68^Ga]­Ga-HBED-CC-PSMA (i.e., PSMA-11) for the imaging of prostate tumors.^34–36^

However, HBED does have a main drawback: it can give rise to multiple isomeric [^68^Ga]­Ga^3+^ complexes, with their distribution being highly sensitive to reaction conditions such as temperature and pH.^36–38^ This is particularly concerning, as the presence of these different isomers in the final radiopharmaceutical formulation may result in distinct pharmacological profiles, potentially influencing the in vivo performance of the radiolabeled compound.

In this study, we introduce a rigidified derivative of HBED, *N*,*N*′-di­(2-hydroxybenzyl)-(1,2-cyclohexanediamine)-*N*,*N*′-diacetic acid (HBCD, Figure 1), where the flexible ethylenediamine fragment of HBED is replaced by a more rigid cyclohexane diamine (DACH) scaffold. This modification is based on the hypothesis that adding steric hindrance will thwart the formation of multiple isomers while preserving a high biological stability of the corresponding [^68^Ga]­Ga^3+^ complex.

Although the structure and preparation of a similar compound (*trans*-[[2-[ethoxycarbonylmethyl-(2-hydroxybenzyl)­amino]­cyclohexyl]-(2-hydroxybenzyl)­amino]­acetic acid) has been claimed in a patent (WO 9744313 A1 ^39^), to date no chemico-physical data are available in literature for HBCD. Hence, we report herein the synthesis and acid–base behavior of HBCD, along with the assessment of its ability to complex Ga^3+^ in aqueous solution and a detailed evaluation of its coordination chemistry. To fully evaluate the potential of HBCD as a chelator for ^68^Ga-based radiopharmaceuticals, we also describe its performance in chelating [^68^Ga]­Ga^3+^ under radiochemical conditions and examine the stability of the resulting [^68^Ga]­Ga^3+^ complex in physiological conditions (phosphate buffered saline and human serum).

## 2 Results and Discussion

### 2.1 Synthesis of HBCD

HBCD was synthesized through a multistep process, as detailed in Figure 2. The synthesis began with the condensation of 1,2-diaminocyclohexane (**1**) and 2-hydroxybenzaldehyde (**2**), to form the imine intermediate (**3**). The latter was then reduced in situ using sodium borohydride, resulting in the formation of *N*,*N*′-di­(2-hydroxybenzyl)-1,2-cyclohexanediamine (**4**). The reaction proceeded to the *N*-alkylation step, where (**4**) was treated with *t*-butyl bromoacetate (**5**), yielding N,N′-di­(2-hydroxybenzyl)-(1,2-cyclohexanediamine)-*N*,*N*-diacetic acid di-*t*-butyl ester (**6**). The final step involved the deprotection of (**6**) using trifluoroacetic acid (TFA), yielding the desired product, N,N′-di­(2-hydroxybenzyl)-(1,2-cyclohexanediamine)-N,N′-diacetic acid (HBCD, **7**), as a TFA salt. The *N*-alkylation reaction represents the limiting step of the overall synthetic strategy being the yield quite low (37%). Many factors could be responsible for the low yield, among them the formation of H-bonds between the ammine and the phenolic groups hampering the SN_2_ reaction and the potential competition of the phenolic oxygen lacking protection.

**Figure 2:**
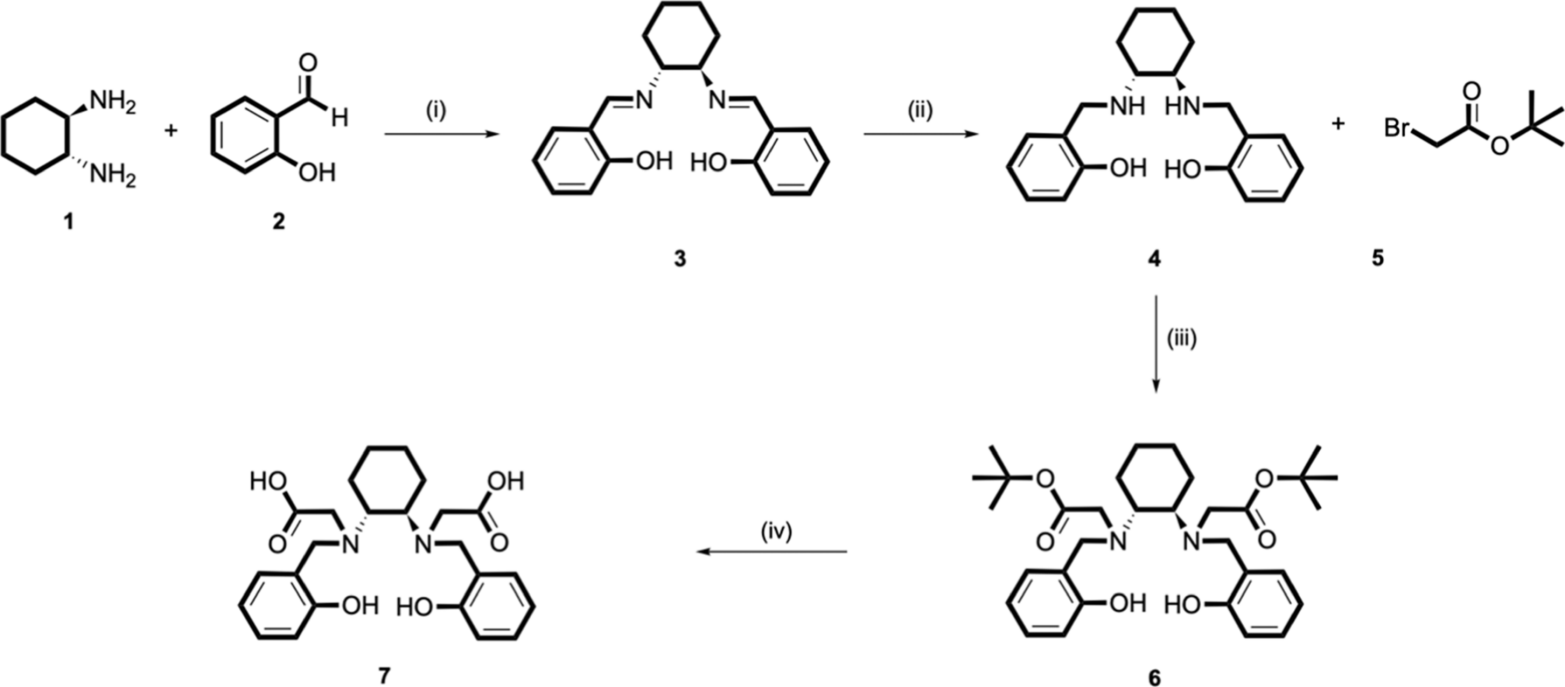
Reaction scheme for the synthesis of HBCD: (i) methanol, reflux, 2.5 h; (ii) NaBH_4_, 0 °C to RT, 2 h, yield 80%; (iii) *N*,*N*-diisopropylethylamine, dimethylformamide, 80 °C, overnight, yield 37%; (iv) TFA, RT, 2 h, yield 99%.

All the intermediates and the final chelator, HBCD, were fully characterized in nonaqueous solvent by nuclear magnetic resonance (NMR) spectroscopy, electrospray ionization mass spectrometry (ESI-MS), and elemental analysis as detailed in Figures S1–S7.

### 2.2 Acidity Constants of HBCD

The metal affinity of a ligand is closely linked to its acid–base properties, as protons can compete with metal ions for interactions at donor sites that exhibit acid–base characteristics. In HBCD, such sites include the tertiary amines of the cyclohexane diamine backbone, the carboxylic groups, and the phenolic oxygens. Therefore, the protonation equilibria of HBCD were investigated in aqueous solution using UV–vis and NMR spectroscopies before evaluating its ability to chelate Ga^3+^.

Under acidic conditions, HBCD exhibits an electronic spectrum with maximum absorption at 275 nm (Figure 3). As the pH increases, the intensity of the band at 275 nm decreases, while two new bands emerge at 241 and 298 nm, with their absorbance progressively increasing. Notably, two isosbestic points at ∼260 and ∼280 nm also appear, indicating the presence of multiple acid–base equilibria. These pH-dependent changes in the electronic spectra allowed the determination of the acidity constants (p*K*_a_) of HBCD, which are summarized in Table 1.

**Figure 3:**
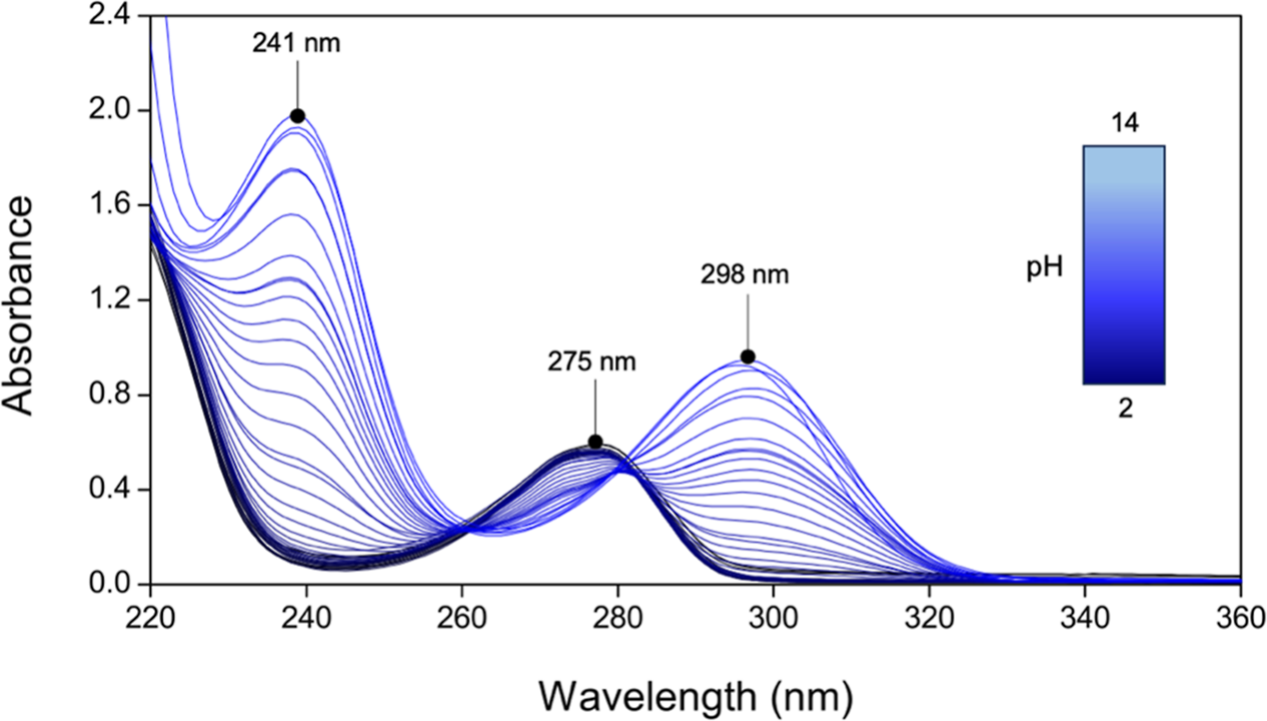
Representative UV–vis spectra of HBCD (L) at different pH (*C*_L_ = 100 μM, *I* = 0.15 M NaCl, *T* = 25 °C).

**Table 1:** Acidity Constants (p*K*_a_) of HBCD at *T* = 25 °C and *I* = 0.15 M NaCl^a^

equilibrium reaction^b^		HBCD (UV–vis)	HBCD (NMR)	HBED^c^
HL^3–^ ⇌ H^+^ + L^4–^	p*K*_a,6_	13.2 ± 0.2	13.5 ± 0.2	12.64
H_2_L^2–^ ⇌ H^+^ + HL^3–^	p*K*_a,5_	11.94 ± 0.03	12.2 ± 0.1	11.03
H_3_L^–^ ⇌ H^+^ + H_2_L^2–^	p*K*_a,4_	9.42 ± 0.04	9.7 ± 0.3	8.34
H_4_L ⇌ H^+^ + H_3_L^–^	p*K*_a,3_	5.20 ± 0.06	4.9 ± 0.4	4.40
H_5_L^+^ ⇌ H^+^ + H_4_L	p*K*_a,2_	^d^	3.1 ± 0.3	2.24
H_6_L^2+^ ⇌ H^+^ + H_5_L^+^	p*K*_a,1_	^d^	1.1 ± 0.2	

a Literature data for HBED are reported for comparison purposes.^40^

b L represents the completely deprotonated form of the ligand. The reported uncertainty was obtained by the fitting procedure and represents one standard deviation unit.

c I = 0.10 M KCl, *T* = 25 °C.^40^

d An average value of p*K*_a,1_ and p*K*_a,2_ equal to 1.7 ± 0.2 was calculated from UV–vis data.

The UV–vis data were complemented by pH-dependent ^1^H NMR measurements, with representative spectra presented in Figure 4. Selected bidimensional spectra used for signal assignments are displayed in Figures S9 and S10.

**Figure 4:**
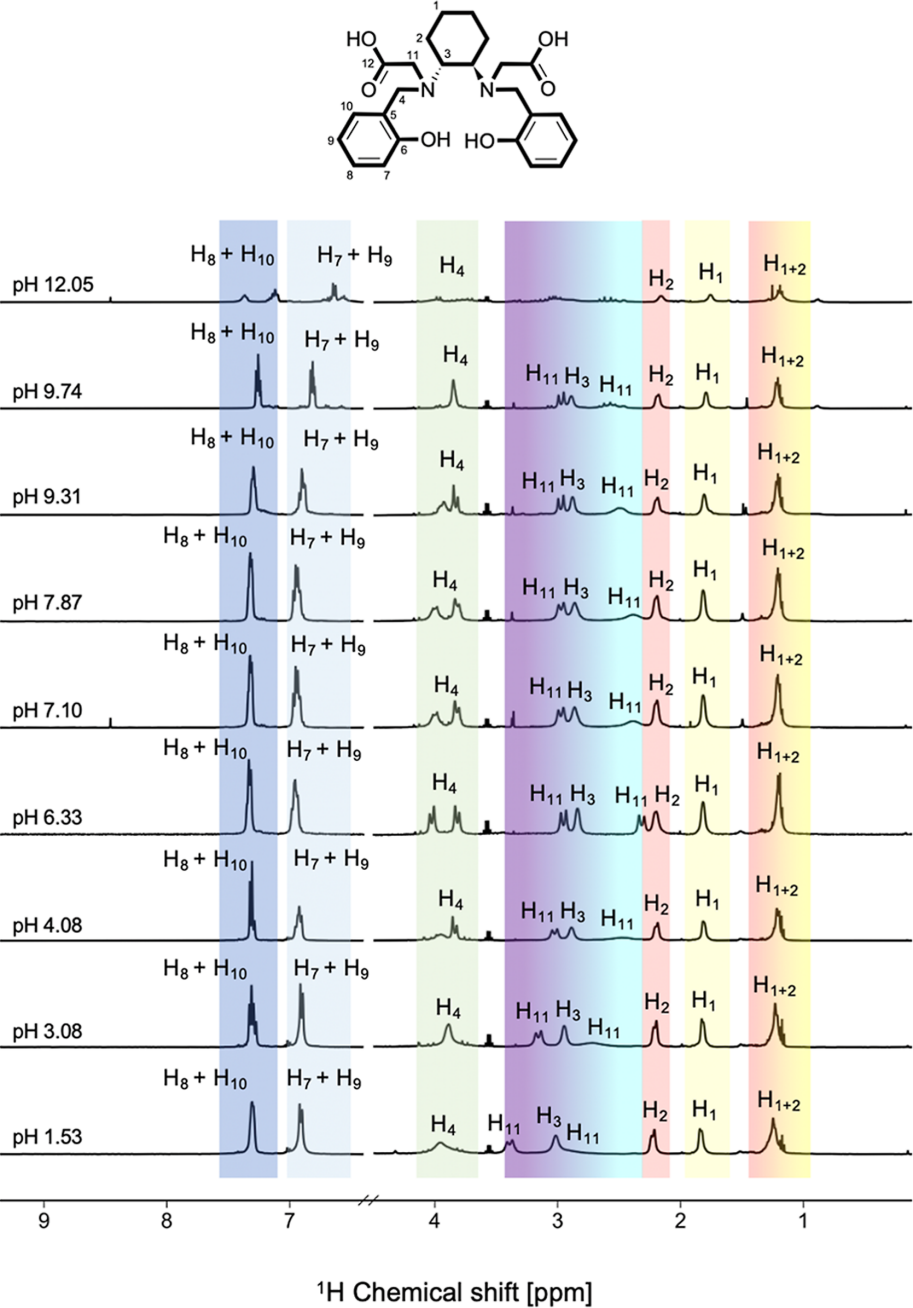
^1^H NMR spectra (600 MHz, D_2_O, *T* = 25 °C, *I* = 0.15 M NaCl) of HBCD at different pH values and signal attributions.

The signals corresponding to the aromatic protons of the phenol-containing pendants (H_7_, H_8_, H_9_, and H_10_—see Figure 4 for atom numbering) remain relatively unchanged in the acidic region. However, as the pH increases (pH > 8), these signals undergo a progressively increasing shielding.

In the nonaromatic region, significant changes in chemical shifts and multiplicity are observed across the investigated pH range, particularly for the protons in α position to the amine and carboxylic groups (H_3_, H_4_, H_11_) due to the occurrence of their deprotonation events (Table 1). For the *N*-bound CH_2_ protons of the phenolic pendant (H_4_), the signal appears as a broad singlet at very acidic pH (δ = 3.95 ppm) and remains unchanged up to pH 3.08. At pH 4.08, the signal begins to split, evolving into two doublets around pH 6.33 (δ = 3.81 ppm and δ = 4.01 ppm, respectively), suggesting that the inversion of the amine N atom is slow on the NMR time scale, likely because of intramolecular hydrogen bonding involving the amine and phenol groups. As the pH increases further, the signal reverts to a singlet (δ = 3.82 ppm). The deshielding-shielding-deshielding pattern is similarly observed for the CH_2_ protons linked to both the N and the COOH groups (H_11_) and for the *N*-bound CH proton of the cyclohexane ring (H_3_).

At pH > 11, a marked change in the fine structure of the spectrum is observed, with the signals approaching coalescence. Notably, at extremely high Na^+^ concentration (pH > 12), the coordination of sodium cation by HBCD could be expected by the signal line broadening suggesting the formation of [NaH_*x*_L]^(*x*−3)^ (H_*x*_L^(*x*–4)^ = HBCD), as previously reported in the literature for similar chelators.^41^

The acidity constants obtained by ^1^H NMR titrations (Table 1) are consistent with those calculated from spectrophotometric titrations, furthermore these data allowed to estimate the two lowest p*K*_a_ values corresponding to the carboxyl groups. The assignment of the other four protonation constants (p*K*_a,3_, p*K*_a,4_, p*K*_a,5_ and p*K*_a,6_) is extremely challenging. For the lead compound HBED, Martell and co-workers suggested the order of the basicity: phenolate > amino > carboxylate groups.^42^ According to NMR data of HBCD, p*K*_a,3_ can reasonably be assigned to one of the tertiary ammine. Once this dissociation occurs, the nitrogen atom can form a hydrogen bond with the phenolic group or with the other amine group. The next dissociation (p*K*_a,4_) can be attributed to the other phenolic moiety that can be then involved in the formation of the hydrogen bond with the protonated ammine. The last two dissociations can tentatively be attributed to the second phenolic group (p*K*_a,5_) and the tertiary amine (p*K*_a,6_).

The speciation diagrams of HBCD, shown in Figure S8, indicates that the physiologically predominant form is H_3_L^–^. Notably, a comparison of the p*K*_a_ of HBCD with those reported in the literature for the parent compound HBED reveals that the former is more basic (Table 1 and Figure S8). This difference likely arises from the structural rigidity of the inserted cyclohexane diamine backbone compared to the more flexible ethylenediamine fragment in HBED. A similar trend has been observed for other chelators upon the incorporation of the same scaffold (e.g., dedpa vs CHXdedpa).^26,43^

### 2.3 Formation Kinetics of Ga^3+^-HBCD

Before the thermodynamic investigation, the formation kinetics of the Ga^3+^-HBCD complexes were qualitatively assessed using ^1^H NMR and UV–vis spectroscopies at different pH and concentrations to determine the time needed to reach equilibrium. Understanding the equilibration conditions is essential for obtaining accurate thermodynamic data and for gaining an initial insight into the binding capacity of the new chelator. Selected representative time-dependent UV–vis and ^1^H NMR spectra of Ga^3+^-HBCD mixtures at different pH are shown in Figures S11 and 5.

**Figure 5:**
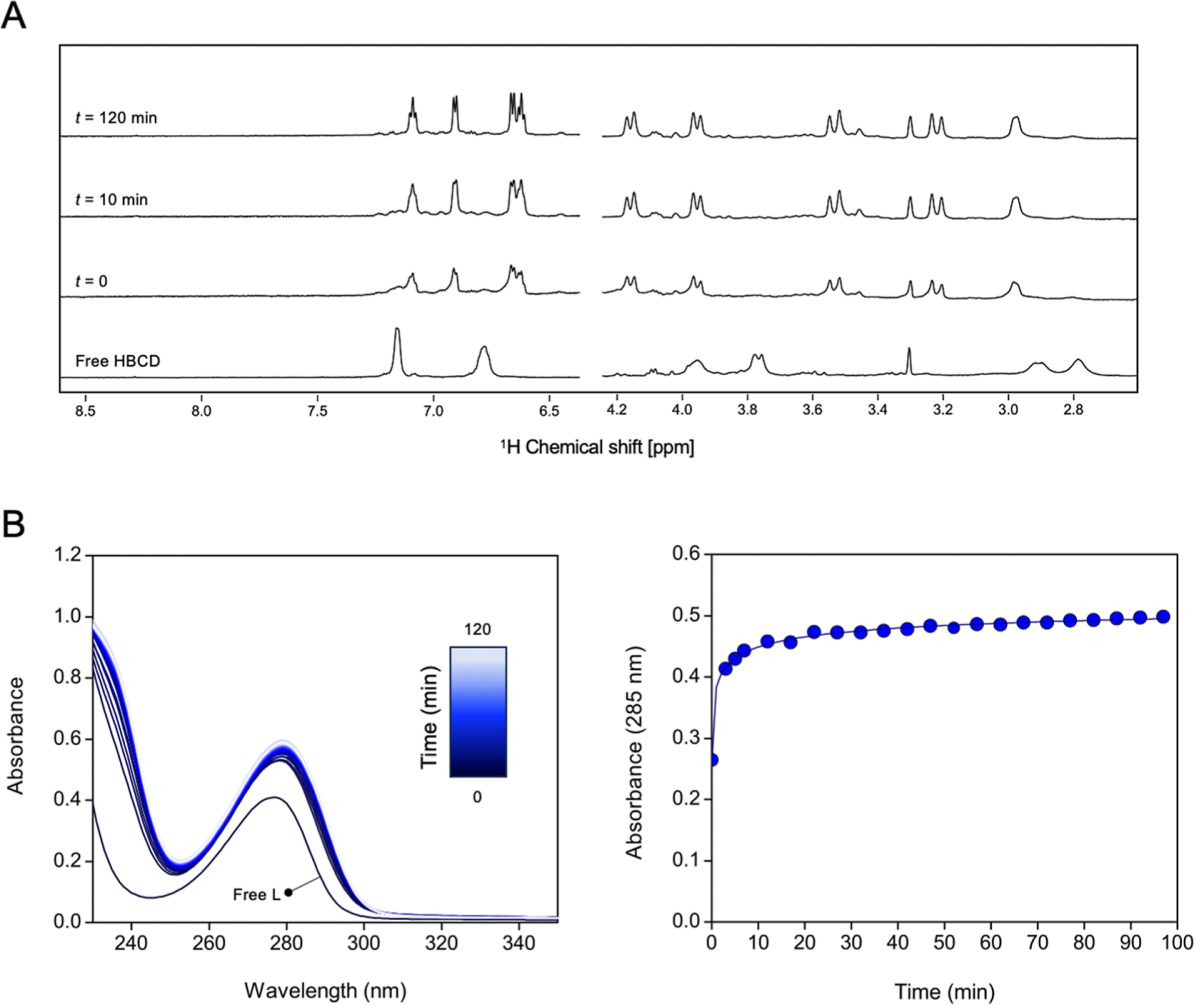
Formation kinetics of Ga^3+^-HBCD at pH 4.5 (acetate buffer, 0.01 M) and *T* = 25 °C: (A) time-dependent ^1^H NMR spectra (600 MHz, D_2_O, *T* = 25 °C, *C*_Ga_ = *C*_L_ = 1 mM. Signals at ppm <2.5 have been omitted due to the presence of higher intensity signals from acetate protons; signal at 3.3 ppm is related to methanol impurities) and (B) time-dependent UV–vis spectra (left) and plot of absorbance at 285 nm vs time (right) (*C*_Ga_ = *C*_L_ = 50 μM).

The formation kinetics were found to be notably slow at highly acidic pH. For example, at pH ∼ 2, no complex formation was observed at room temperature after 24 h, as the ^1^H NMR spectra remained identical to that of the free HBCD (Figure S11). Complexation only occurred after heating (*T* = 80 °C, overnight), suggesting that the binding process is very slow under these conditions. At pH ∼ 4.5, the complexation was nearly instantaneous at mM metal and ligand concentrations, while at lower concentrations (50 μM), the reaction rate was slower, taking approximately 1 h to reach completion, as shown from ^1^H NMR and UV–vis data, respectively (Figure 5).

The pH dependence of the complexation rate can be attributed to the increasing protonation of the donor groups as the pH decreases (Table 1). These protonation events reduce the ability of HBCD to complex the metal ion due to the Coulombic repulsions between the two positively charged species.

### 2.4 Thermodynamic Stability of Ga^3+^-HBCD

The thermodynamic stability of Ga^3+^-HBCD was evaluated by UV–vis and ^1^H NMR spectroscopies. Due to the slow complexation kinetics observed at highly acidic pH values, in-batch titrations were conducted, and heating was applied to speed up the attainment of equilibrium under these conditions. Representative pH-dependent ^1^H NMR and UV–vis spectra are reported in Figures S12 and S13, respectively. The overall stability constants (log β) of Ga^3+^-HBCD are provided in Table 2 while the corresponding distribution diagram is shown in Figure 6 in comparison with HBED.

**Table 2:** Overall Stability Constants (Log β) of the Ga^3+^ Complexes Formed by HBCD (*T* = 25 °C and *I* = 0.15 M NaCl) and HBED and pGa^3+^ Values^40^

equilibrium reaction^a^	log β	
	HBCD	HBED ^(^ ^b^ ^)^
Ga^3+^ + H^+^ + L^4–^ ⇌ [GaHL]	41.2 ± 0.1	40.81
Ga^3+^ + L^4–^ ⇌ [GaL]^−^	39.2 ± 0.1	38.51
pGa^3+^ ^c^	27.0	29.6

a L represents the completely deprotonated form of the ligand. The reported uncertainty was obtained by the fitting procedure and represents one standard deviation unit.

b Data from ref 40.

c pGa^3+^ calculated at pH 7.4, *C*_L_ = 10 μM and *C*_Ga_ = 1 μM; Hydrolysis constants of [Ga­(OH)_*r*_]^(3–*r*)^ species (1 ≤ *r* ≤ 4) were taken from ref 44

**Figure 6:**
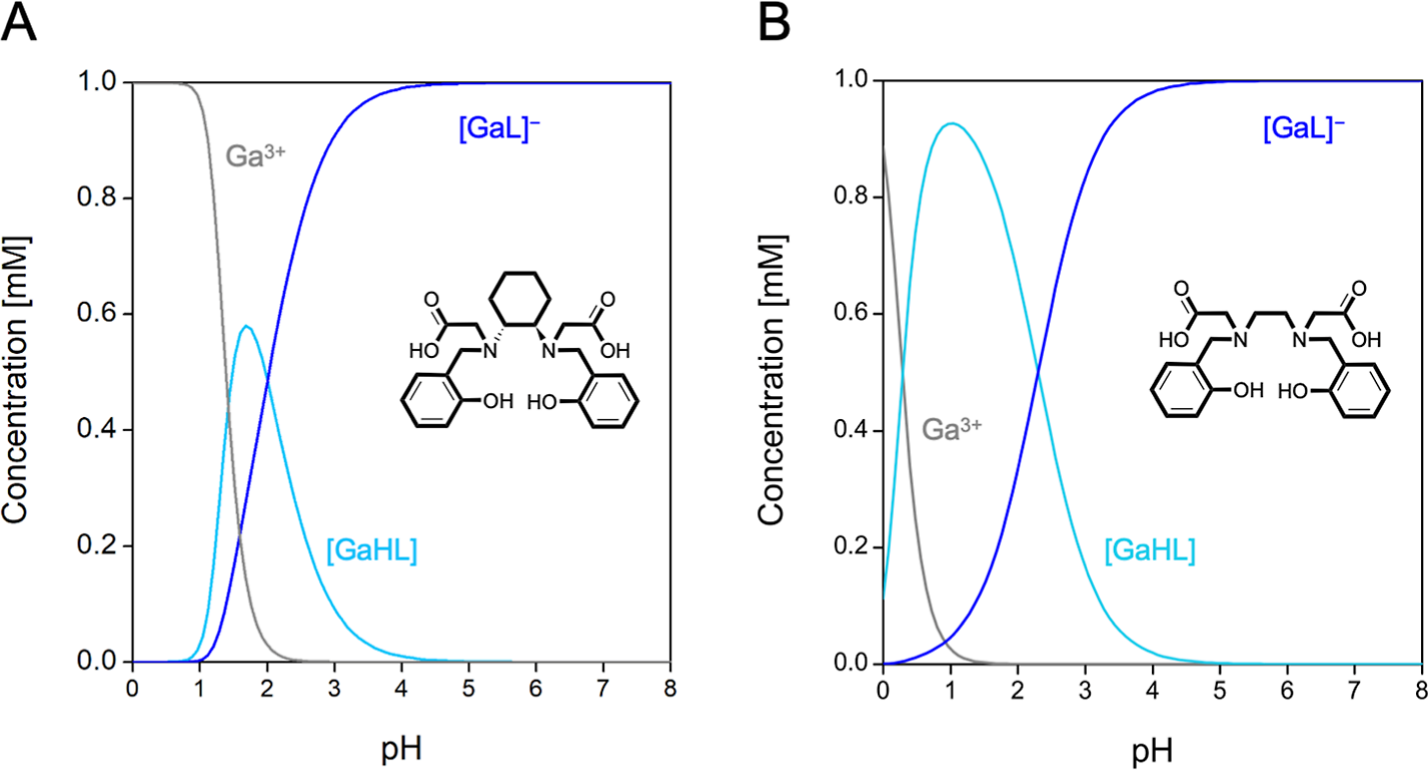
Distribution diagram of (A) Ga^3+^-HBCD and (B) Ga^3+^-HBED (*C*_Ga_ = *C*_L_ = 1 mM).

At extremely low pH (<0.5), only the signals belonging to free HBCD are observed. At pH > 1, the ^1^H NMR spectra exhibit clear differences compared to those of free HBCD, indicating that Ga^3+^ complexation occurs within this pH range. At pH > 3, the spectra remain unchanged, reflecting the presence of the fully deprotonated complex [GaL]^−^. At pH < 3, additional signals emerge, corresponding to the formation of the monoprotonated complex [GaHL]. The 1:1 Ga-to-HBCD complex stoichiometry was further confirmed via mass spectrometry (Figure S14).

Importantly, the inclusion of the DACH moiety does not affect the speciation, as the Ga^3+^-HBCD speciation remains identical to that of HBED.^40^

Regarding the UV–vis data, the electronic spectra of Ga^3+^-HBCD consistently differ from those of the free ligand (compare Figure 3 with Figure S13), providing clear evidence of the complexation. However, the spectra show only slight variations with changes in pH, primarily limited to a minor shift in the absorbance maximum. As a result, the technique provided predominantly qualitative data on the complexation event.

To compare the chelating abilities of HBCD and HBED, the pGa^3+^ values (pGa^3+^ = −log­[Ga^3+^]_free_) were calculated under physiological conditions (pH = 7.4, *C*_L_ = 10 μM and *C*_Ga_ = 1 μM). This parameter accounts for both ligand basicity and metal-ion hydrolysis, with higher pGa^3+^ values indicating greater complex stability under the given conditions.^45^ The resulting values are provided in Table 2.

pGa values together with speciation diagrams suggest that HBCD forms a Ga^3+^ complex with an extremely high thermodynamic stability in physiological conditions, though lower than that of Ga^3+^-HBED. This outcome could be related to the presence of the DACH moiety which increases the rigidity of the HBCD chelator—enhancing kinetic inertness but potentially compromising stability. Additionally, the lack of the first dissociation constant of HBED might affect the calculations.

When related to other state-of-the-art chelators, such as the widely used DOTA (pGa^3+^ = 18.5 under the same conditions^26^), HBCD demonstrates an exceptional superior stability in its Ga^3+^ complex. Furthermore, HBCD forms a Ga^3+^ complex with greater stability compared to transferrin (pGa^3+^ = 20.3 under the same conditions^26^), one of the primary in vivo competitors for Ga^3+^. This is particularly significant, as transferrin can induce demetalation, potentially leading to undesirable radioactivity accumulation in nontumor tissues. These results highlight the promising potential of HBCD and strongly support the need for further testing under radiochemical and biological conditions.

### 2.5 Ga^3+^-HBCD Structure in Aqueous Solution

NMR spectroscopy also provides insight into the structure of Ga^3+^-HBCD in aqueous solution. Figure 7 shows the ^1^H and ^13^C NMR spectra of [GaL]^−^, comparing them with those of the free ligand, both of which share the same total net charge (−1). Bidimensional spectra of [GaL]^−^, used to support the signal attributions, are presented in Figures S15 and S16.

**Figure 7:**
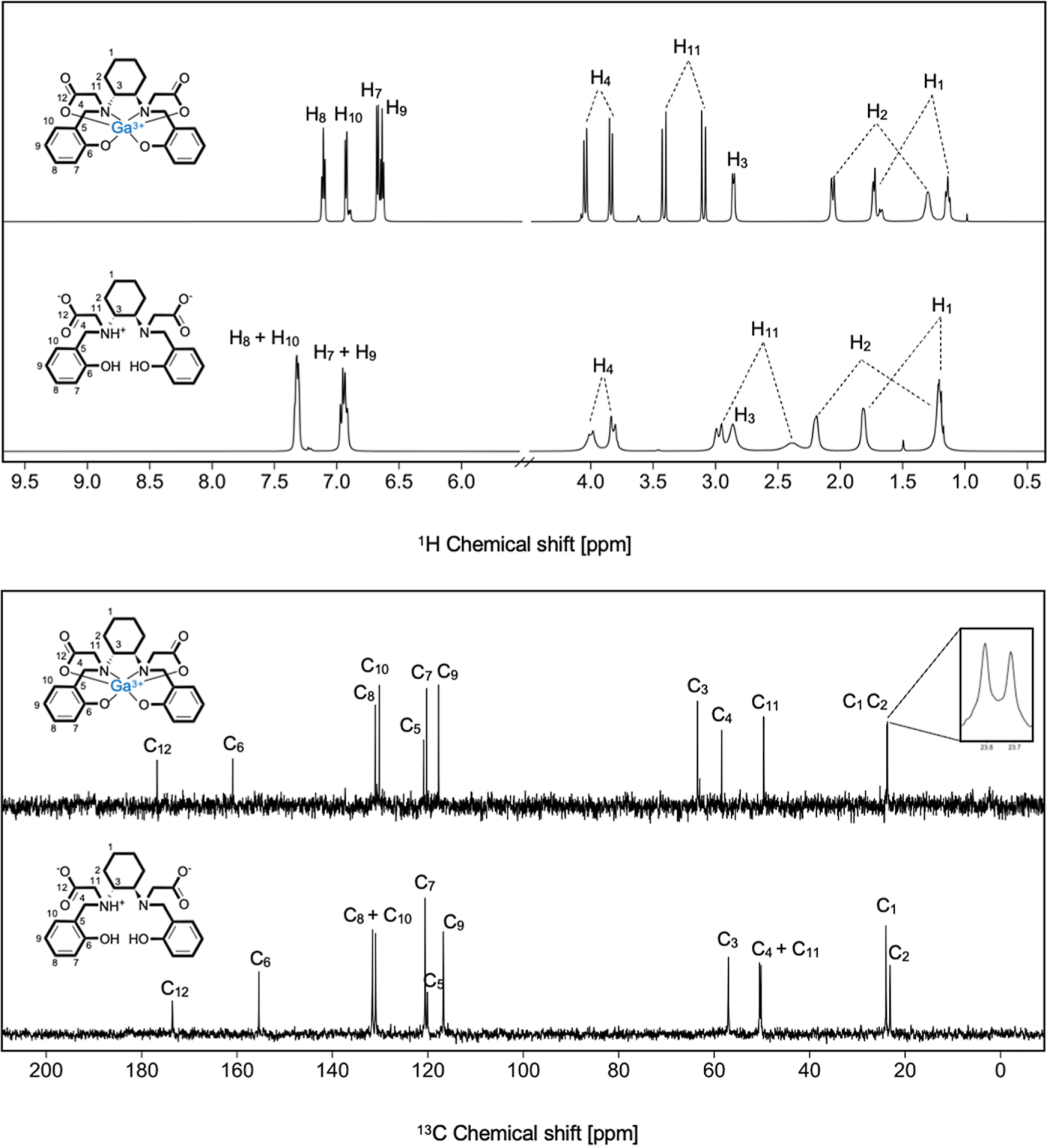
Comparison of ^1^H NMR (up) and ^13^C NMR spectra (down) of [GaL]^−^ and free HBCD (600 MHz, D_2_O, *T* = 25 °C).

In the presence of the metal ion, all ^1^H/^13^C NMR signals show variations in chemical shifts and/or changes in their fine structure. Considering these variations, alongside the coordination preference of Ga^3+^, it is likely that all donor atoms are involved in the metal coordination sphere, forming a N_2_O_4_ environment.

In [GaL]^−^, the aromatic ^1^H resonances appear at distinct chemical shifts, indicating a significant alteration in the electronic environment upon Ga^3+^ coordination. In the free ligand, these protons originally resonated in pairs at identical chemical shifts.

All the methylene protons are nonchemical shift equivalent, not only those of the DACH moiety (H_1_ and H_2_) which assignment of the axial and equatorial protons is straightforward due to the strong ^3^ *J*_ax‑ax_ and weak ^3^ *J*_ax‑eq_ in addition to the geminal scalar coupling. This diasterotopic splitting was (partially) previously observed in the free ligand and was attributed to the presence of the cyclohexane diamine ring, which induces magnetic nonequivalence in these protons. This effect is preserved and further amplified upon coordination with the metal center, likely due to an increased degree of structural rigidity.

Additionally, all signals are sharp, suggesting that the metal coordination leads to the formation of a rigid hexacoordinated structure. Contrary to what was previously reported in the literature for Ga^3+^-HBED, no isomers are detected.^36–38^ Indeed, the ^1^H NMR signals of [GaL]^−^ can be interpreted as representing a single, electronically unique isomer in solution. This is further confirmed by the ^13^C NMR spectrum, where a single resonance for each pair of equivalent carbon atoms on both sides of the coordinated ligand is visible.

No spectral variations were observed with temperature changes, reinforcing the hypothesis of high rigidity in the complex (data not shown, as the spectra are identical to that reported in Figure 7).

Interestingly, the complex and irregular ^1^H NMR signals characteristic of [Ga­(HL)] (Figure S12) indicate that the protonation significantly alters the electronic environment of the Ga^3+^ complex when compared to [GaL]^−^. This pattern suggests the formation of a highly asymmetric complex in which the chelator acts as a pentadentate ligand and probably the sixth coordination site of Ga^3+^ is occupied by a water molecule.

### 2.6 DFT Calculations

Density functional theory (DFT) calculations were performed on Ga^3+^-HBCD complexes to gain additional insights into their structures.

For the physiologically dominant species, i.e. [GaL]^−^, the ligand adopts its most stable form by coordinating to Ga^3+^ through six donor groups (Table 3). Besides the two amines, always in *cis* position, the remaining phenolate (PhO^–^) and carboxylic (OAc^–^) groups can be arranged in three different geometries to achieve an octahedral coordination. Specifically, both the OAc^–^ and the PhO^–^ groups may be positioned in *cis* relative to each other (isomer A, Figure 8), or one of them could occupy a *trans* position (isomer B or C, Figure 8). As reported in Table 3, the form with *cis* PhO^–^ and *trans* OAc^–^ is significantly more stable, suggesting that this configuration is the preferred. For completeness, the geometries were optimized with TPSSTPSS functional, D3 dispersion correction, and SAS for the solute cavity.^46,47^ The resulting relative energies (Table S3) are consistent with those calculated with B3LYP. In addition, the *C*2 symmetry of this isomer aligned well with the outcomes driven from ^1^H NMR spectra. The optimized structure of this species is depicted in Figure 9 while the structures of the other isomers are provided in Figure S17.

**Table 3:** DFT-Calculated (B3LYP) Relative Energies for Possible [GaL]^−^ Configurations

configuration			relative energy (kcal/mol)
6 donors	A	PhO *cis*, COO *cis*	5.2
	B	PhO *cis*, COO *trans*	0.0
	C	PhO *trans*, COO *cis*	15.6
5 donors + H_2_O	D	COO *trans*	13.2

**Figure 8:**
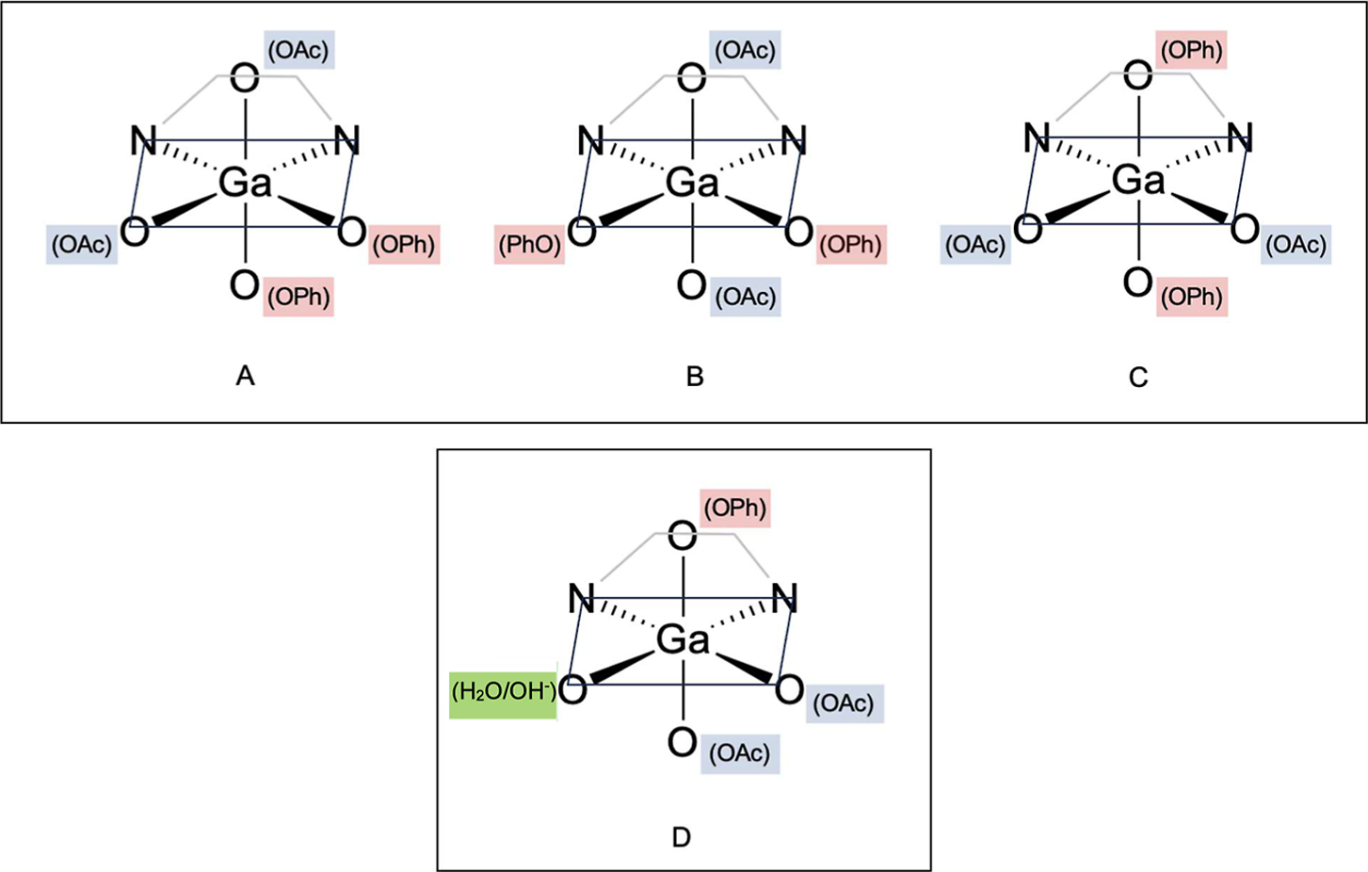
Schematic representations of possible [GaL]^−^ isomers.

**Figure 9:**
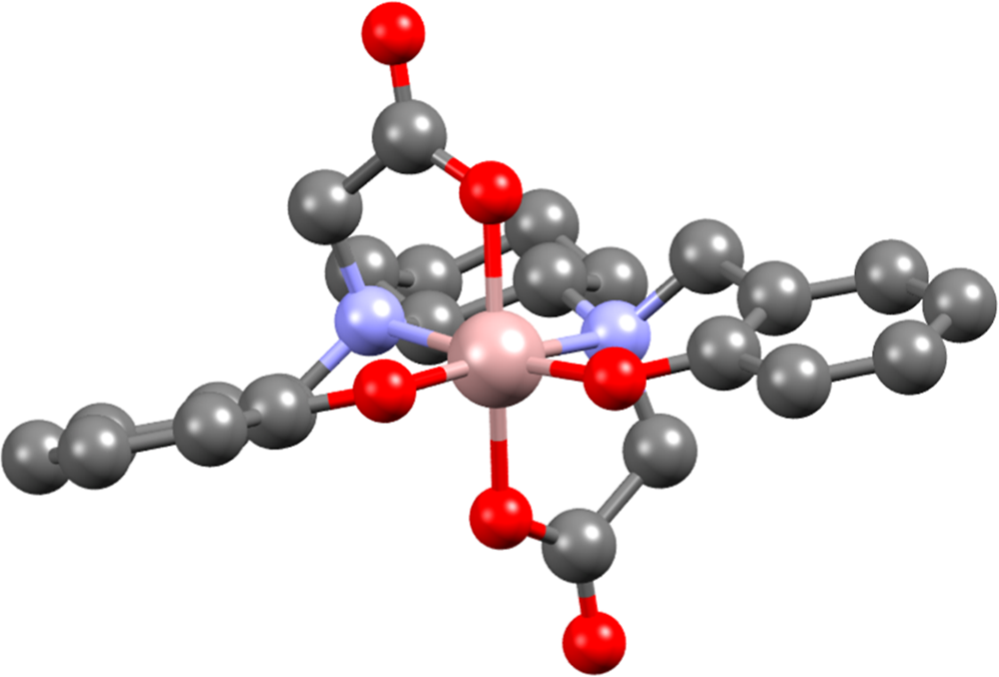
DFT-optimized structure of 6-coordinated *cis*–*trans* [GaL]^−^.

The possibility of a 6-coordinated complex where the sixth coordination site is occupied by an explicit H_2_O molecule and one deprotonated PhO^–^ arm remains unbound to the metal center, was also considered. In this case, it is favorable to transfer a proton from the coordinating water molecule to the phenolate (Figures 8 and S17). However, this configuration results in a significantly higher energy, indicating that it is not energetically favorable (Table 3).

^1^H NMR and ^13^C NMR spectra for the 6-coordinated [GaL]^−^ complex were simulated using DFT, accounting for all possible configurations. The resulting spectra, shown in Figure S18, are accompanied by the corresponding numerical values in Tables S1 and S2.

Although the simulated spectra align well with the experimentally observed resonances, the spectra of the different configurations are nearly identical, making it difficult to determine the most stable species based solely on the comparison of theoretical and experimental NMR data. Therefore, the assumption that the hexacoordinated *cis*–*trans* [GaL]^−^ complex is the most likely structure in solution is only based on the energetic calculations described above.

The structure of the protonated Ga^3+^ complex, i.e. [GaHL], was also investigated. However, the computational results are less definitive in this case, as the relative energies depend on the number of explicitly considered hydrogen bonds in the calculation, and the energy differences among the possible geometries are not significant (<1–2 kcal/mol).

### 2.7 Radiolabeling with Gallium-68

Radiolabeling with generator-produced [^68^Ga]­Ga^3+^ was conducted to evaluate the ability of HBCD to trap this radiometal under highly diluted radiochemical conditions. For comparison, parallel radiolabeling experiments were performed using HBED.

Several parameters were tested for their effect on radiochemical incorporation (RCI), including ligand concentration (10^–4^ M ≤ [L] ≤ 10^–9^ M), temperature (RT and 90 °C), and pH (pH 3, 4.5, and 7). The impact of reaction time (5 and 15 min) was also assessed. Longer reaction times were not considered due to the relatively short half-life of [^68^Ga]­Ga^3+^. The results are presented in Figures 10 and 11 and S19.

**Figure 10:**
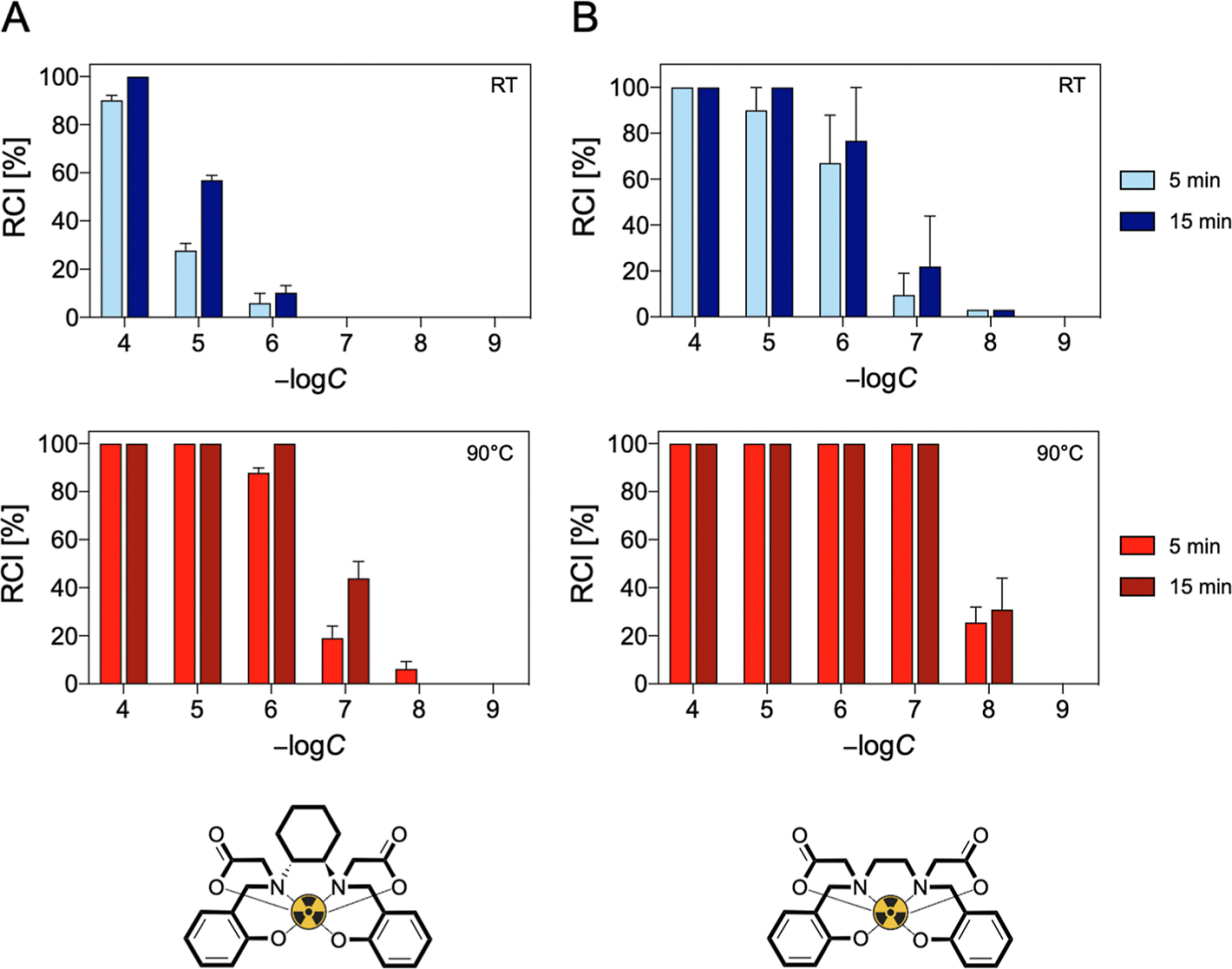
Concentration-, time- and temperature-dependent [^68^Ga]­Ga^3+^ RCIs of (A) HBCD and (b) HBED at pH 4.5.

**Figure 11:**
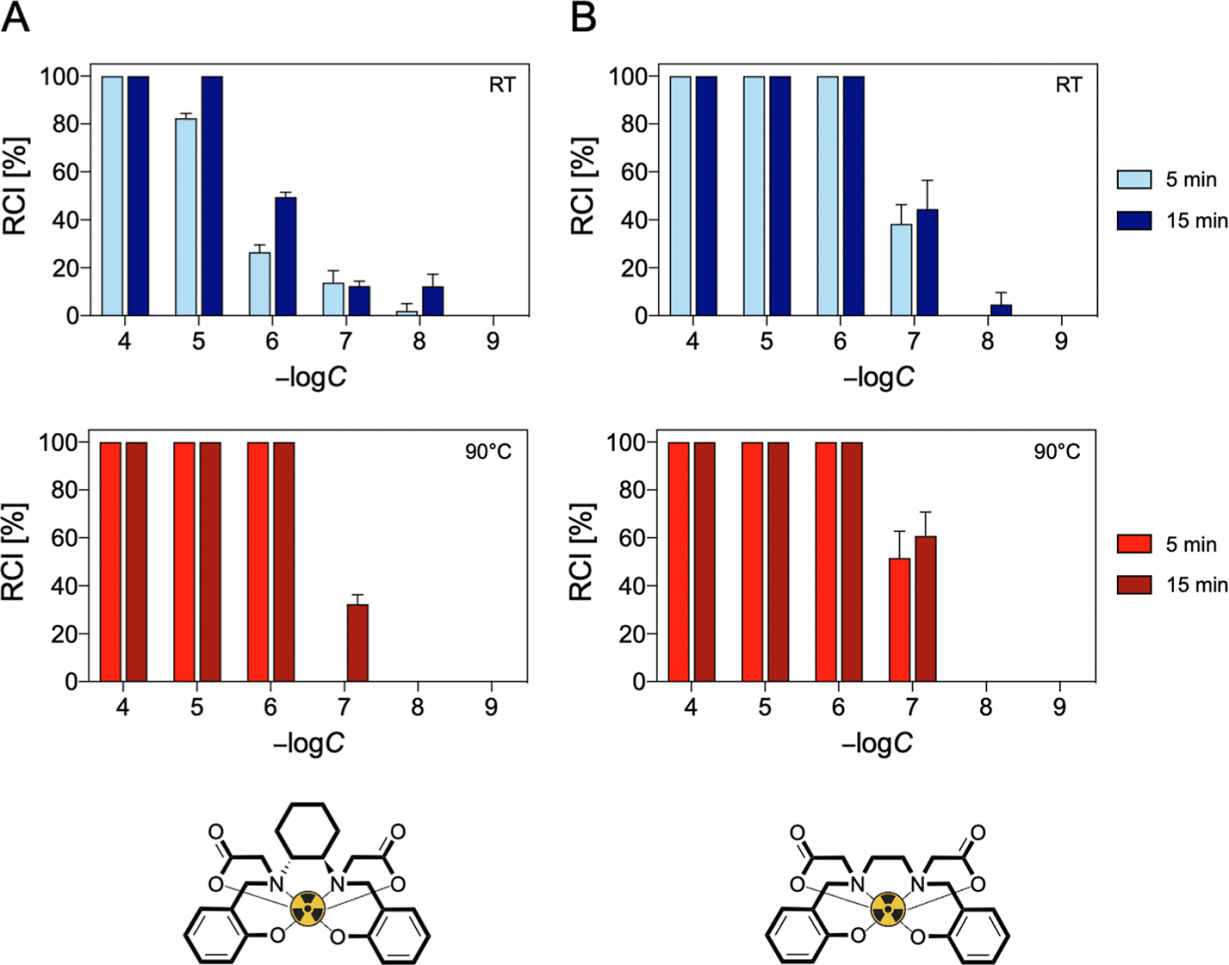
Concentration-, time- and temperature-dependent [^68^Ga]­Ga^3+^ RCIs of (A) HBCD and (b) HBED at pH 7.

At pH 3 and room temperature, HBCD was unable to quantitatively chelate [^68^Ga]­Ga^3+^ under any of the tested conditions, while HBED achieved quantitative [^68^Ga]­Ga^3+^ chelation at 10^–4^ M within 5 min. Heating significantly influenced the RCI for both chelators. At *T* = 90 °C, HBCD displayed quantitatively RCI at 10^–4^ M in 5 min, whereas HBED performed better, achieving quantitative chelation down to 10^–7^ M.

Increasing the pH from 3 to 4.5 improved the RCI for both chelators. Quantitative RCI was achieved with HBCD at 10^–4^ M after 15 min at RT, while HBED achieved the same yield at 1 order of magnitude lower concentration (10^–5^ M). Heating further enhanced the radiometal incorporation, allowing both HBCD and HBED to quantitatively incorporate [^68^Ga]­Ga^3+^ at lower concentrations than at ambient temperature: 10^–6^ M for HBCD (15 min) and 10^–7^ M for HBED (5 min).

At neutral pH, HBCD achieved quantitative RCI at 10^–5^ M after 15 min at RT, while HBED quantitatively chelated [^68^Ga]­Ga^3+^ at 10^–6^ M in 5 min. At *T* = 90 °C, no major differences in the performance of the two chelators can be observed, both reaching quantitative radiometal binding at 10^–6^ M within 5 min.

The general increase in RCI observed for both chelators with rising pH can be attributed to the greater deprotonation of the ligands, which promotes the metal complexation and enables quantitative binding at progressively lower concentrations.

When comparing the two ligands, HBED outperforms HBCD at room temperature. This could be attributed to the presence of the cyclohexane diamine group in HBCD, which introduces rigidity and affects the reaction rate. However, at *T* = 90 °C and pH > 4, both chelators exhibit nearly identical behavior. This is likely due to the temperature increase, which provides sufficient thermal energy to overcome the kinetic constraints observed at room temperature.

### 2.8 Toward Clinical Applications: [^68^Ga]­[Ga­(HBCD)]^−^ Stability in Biological Media

The stability of [^68^Ga]­[Ga­(HBCD)]^−^ was evaluated under conditions simulating the biological environment, including phosphate buffered saline (PBS) and human serum. As shown in Figure 12, the complex remained stable for up to 2 h in both media. A similar stability profile was observed for [^68^Ga]­[Ga­(HBED)]^−^. This confirms our initial hypothesis that the addition of the cyclohexane diamine moiety does not degrade/weaken the integrity of the complex, but rather hinders the formation of multiple isomers.

**Figure 12:**
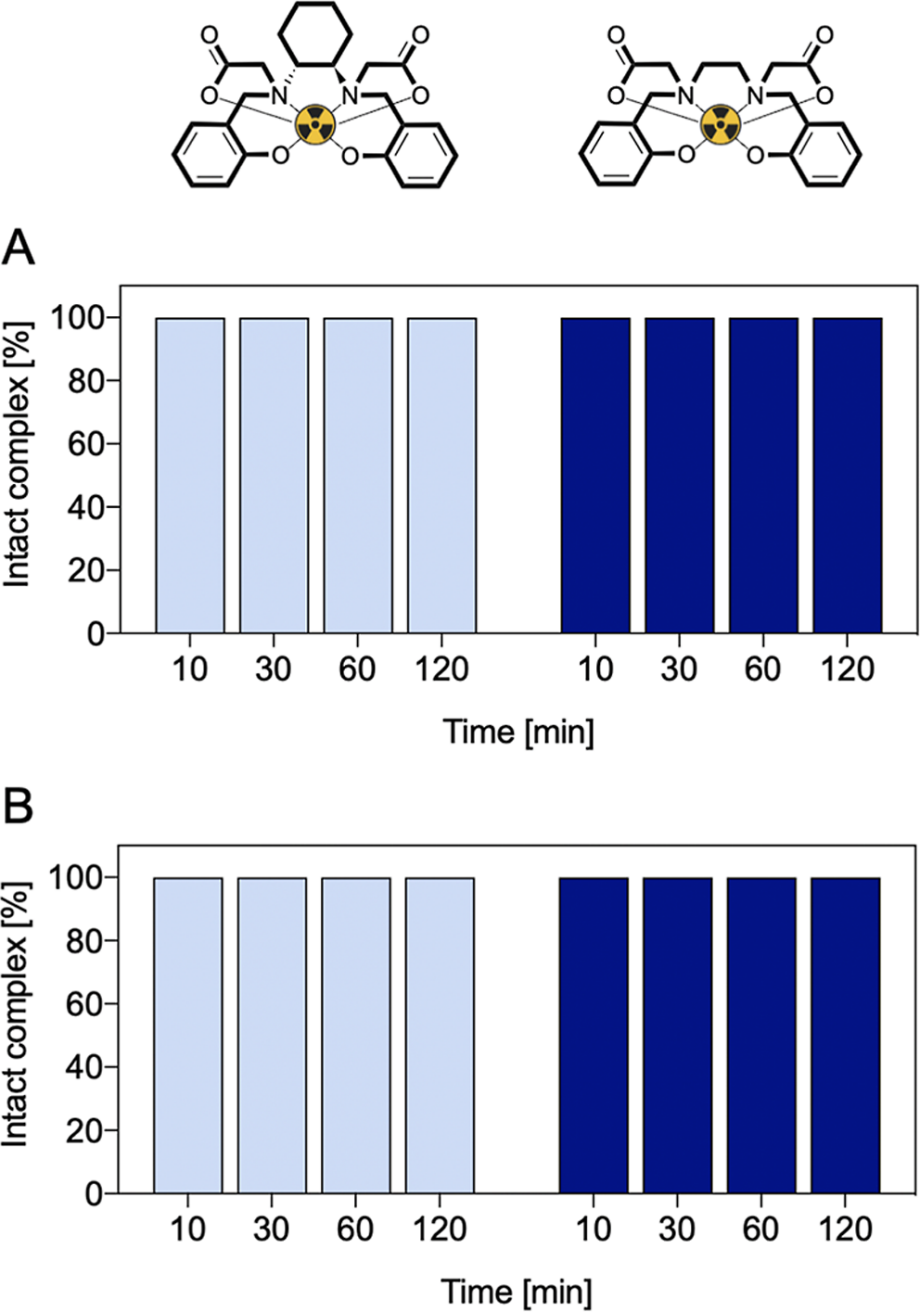
Stability of [^68^Ga]­[Ga­(HBCD)]^−^ and [^68^Ga]­[Ga­(HBED)]^−^ in (A) PBS and (B) human serum at *T* = 37 °C.

## 3 Experimental Section

### 3.1 General

All chemicals were obtained from commercial suppliers and used as received without further purification. All solutions were prepared with ultrapure water (18.2 MΩ/cm) obtained from a Milli-Q Millipore system.

Flash column chromatography was performed using silica gel (60 Å, 230–400 mesh, 40–63 μm, Sigma-Aldrich) and appropriate mobile phases, as described in the following section.

NMR spectra were recorded on a Bruker AVANCE AMX 400 spectrometer (400.12 MHz ^1^H; 100.13 MHz ^13^C) or a Bruker AVANCE AMX 600 spectrometer equipped with a CryoProbe BBO H&F 5 mm in inverse detection (600.13 MHz ^1^H, 150.13 MHz ^13^C). Chemical shifts (δ) are reported in parts per million (ppm), referenced to the residual solvent peak in organic solvents or 3-(trimethylsilyl)­propionic acid sodium salt (TSP) in D_2_O. Coupling constants (*J*) are given in hertz (Hz). Mass spectrometry (MS) was performed on an Agilent 6300 Ion Trap LC–MS system with an electrospray ionization (ESI) interface. Elemental analyses were carried out using a Thermo Scientific FLASH 2000 CHNS Analyzer. UV–vis spectra were recorded on a JASCO V-770 UV/vis/NIR spectrophotometer within the 200–500 nm spectral range, using quartz cells (1 cm optical path).

No uncommon hazards were noted during the executions of experiments.

### 3.2 Synthesis

#### 3.2.1 *N*,*N*′-Di­(2-Hydroxybenzyl)-1,2-cyclohexanediamine

To a solution of 1,2-diaminocyclohexane (497 mg, 4.18 mmol, 1.00 equiv) in methanol (30 mL), 2-hydroxybenzaldehyde (872 μL, 8.36 mmol, 2.00 equiv) was added, and the resulting mixture was refluxed for 2 h under Ar atmosphere. The mixture was cooled on an ice bath and NaBH_4_ (649 mg, 17.16 mmol, 4.11 equiv) was added. The resulting solution was stirred at room temperature for 2 h. The reaction was quenched by slowly adding Milli-Q water (20 mL), and the solvent was removed under reduced pressure, yielding a colorless oil. The oil was extracted with dichloromethane (3 × 20 mL), and the combined organic phases were washed with a saturated NaCl aqueous solution (2 × 20 mL), and then dried over anhydrous MgSO_4_. The solvent was removed under reduced pressure, resulting in a transparent oil. The oil was triturated with several washes in ethyl ether, and the residual solvent was removed using a mechanical pump, giving a white powdery solid. This procedure was repeated twice, resulting in the isolation of *N*,*N*′-di­(2-hydroxybenzyl)-1,2-cyclohexanediamine (1.0917 g, 3.35 mmol, 0.8 eq, yield 80%). ^1^H NMR (CDCl_3_, 400 MHz, *T* = 25 °C): δ (ppm): 7.16 (td, 2H, H_8_); 6.97 (dd, 2H, H_10_); 6.87 (dd, 2H, H_7_); 6.78 (td, 2H, H_9_); 4.04 + 3.93 (m, 4H, H_4_); 2.48 (m, 2H, H_3_); 2.18 (m, 2H, H_2_); 1.72 (m, 2H, H_1_); 1.23 (m, 4H, H_1_ + H_2_). ^13^C NMR (CDCl_3_, 400 MHz, *T* = 25 °C): δ (ppm): 128.86 (C_10_); 119.52 (C_9_); 129.20 (C_8_); 116.70 (C_7_); 157.64 (C_6_); 122.61 (C_5_); 49.12 (C_4_); 59.60 (C_3_); 36.30 (C_2_); 24.31 (C_1_). ESI-MS *m*/*z*: [M + H]^+^ 327.1 (found); 327.21 (calcd for C_20_H_27_N_2_O_2_^+^).

#### 3.2.2 *N*,*N*′-Di­(2-Hydroxybenzyl)-(1,2-cyclohexanediamine)-*N*,*N*-diacetic Acid di-*t*-butyl Ester

*N*,*N*′-Di­(2-hydroxybenzyl)-1,2-cyclohexanediamine (996.67 mg, 3.05 mmol, 1 equiv) was dissolved in dimethylformamide (8 mL). *N*,*N*-Diisopropylethylamine (2.2 mL, 12 mmol, 3.9 equiv) was added under an Ar atmosphere. A solution of *t*-butyl bromoacetate (0.942 mL, 6.42 mmol, 2.1 equiv) in dimethylformamide (8 mL) was added dropwise over 30 min. After the addition was complete, the mixture was stirred at *T* = 80 °C overnight. The product was purified through flash column chromatography on silica gel using a gradient of petroleum ether to petroleum ether/ethyl acetate 75/25 as the mobile phase. The fractions containing the product were collected, and the solvent was removed under reduced pressure to obtain *N*,*N*′-di­(2-hydroxybenzyl)-(1,2-cyclohexanediamine)-*N*,*N*-diacetic acid di-*t*-butyl ester as a white solid (644 mg, 1.14 mmol, 0.37 eq, yield 37%). ^1^H NMR (CDCl_3_, 600 MHz, *T* = 25 °C): δ (ppm): 9.52 (s br, 2H, H_11_); 7.20 (td, 2H, H_8_); 6.90 (dd, 2H, H_10_); 6.88 (dd, 2H, H_7_); 6.76 (td, 2H, H_9_); 3.90 + 3.56 (dd, 4H, H_4_); 3.14 + 2.82 (dd, 4H, H_12_); 2.51 (d, 2H, H_3_); 1.98 (d, 2H, H_2_); 1.64 (d, 2H, H_1_); 1.53 (s, 18H, H_15_); 0.80–1.03 (m, 4H, H_1_ + H_2_). ^13^C NMR (CDCl_3_, 600 MHz, *T* = 25 °C): δ (ppm): 172.44 (C_13_); 157.68 (C_6_); 129.92 (C_10_); 129.34 (C_8_); 122.62 (C_5_); 118.96 (C_9_); 116.59 (C_7_); 82.22 (C_14_); 57.79 (C_3_); 53.62 (C_4_); 50.96 (C_12_); 28.29 (C_15_); 26.10 (C_2_); 25.59 (C_1_). ESI-MS: *m*/*z* [M + H]^+^: 555.10 (found); 555.34 (calc. for C_32_H_47_N_2_O_6_^+^). Elemental analysis: % C = 69.18, % H = 8.54; % N = 5.01 (found); % C = 69.29, %H = 8.36; % N = 5.05 (calc. for C_32_H_46_N_2_O_6_).

#### 3.2.3 *N*,*N*′-Di­(2-Hydroxybenzyl)-(1,2-cyclohexanediamine)-*N*,*N*′-diacetic Acid

TFA (700 μL, 9.14 mmol, 72.8 equiv) was added to *N*,*N*′-di­(2-hydroxybenzyl)-(1,2-cyclohexanediamine)-*N*,*N*-diacetic acid di-*t*-butyl ester (69.59 mg, 0.125 mmol, 1 equiv). The mixture was stirred at room temperature for 2 h. The TFA was then removed under reduced pressure, yielding a colorless, viscous oil. This oil was triturated with diethyl ether (2 × 4 mL), and the residual solvent was removed using a mechanical pump. The product was obtained as a white solid (43 mg, 0.064 mmol, 0.51 eq, yield 99%). ^1^H NMR (CD_3_OD, 600 MHz, *T* = 25 °C): δ (ppm): 7.01 (td, 2H, H_8_); 6.95 (d, 2H, H_10_); 6.72 (d, 2H, H_7_); 6.55 (t, 2H, H_9_); 4.00 (s, 2H, H_4_); 3.13 (s. Two H, H_4_); 2.57 (d, 6H, H_11_ + H_3_); 2.04 (d, 2H, H_2_); 1.65 (s, 2H, H_1_); 0.92 (m, 2H, H_1_ + H_2_). ^13^C NMR (D_2_O, 600 MHz, *T* = 25 °C): δ (ppm): 173.58 (C_12_); 155.43 (C_6_); 131.59 + 130.95 (C_8_ + C_10_); 120.60 (C_7_); 120.12 (C_5_); 116.77 (C_9_); 57.05 (C_3_); 50.51 + 50.22 (C_4_ + C_11_); 24.00 (C_1_); 23.15 (C_2_). ESI-MS *m*/*z*: [M + H]^+^ 443.1 (found); 443.22 (calcd for C_24_H_31_N_2_O_6_^+^).

### 3.3 Ga^3+^-HBCD Coordination Chemistry

#### 3.3.1 General

HBCD stock solutions were prepared by dissolving a weighed amount of ligand directly in Milli-Q water to achieve a concentration of approximately 1 mM. The solubility of HBCD in water depends on pH: minimal values occur at acidic pH, where the noncharged form predominates. To enhance solubility, small amount of NaOH were added, and the solution was sonicated for 15 min. The stability of HBCD at room temperature was confirmed by NMR, ESI-MS and UV–vis measurements conducted over the course of up to one month following solution preparation.

Ga^3+^ stock solutions were preprepared from analytical-grade salt and standardized using ICP–MS (1–10 mM). The ionic strength (*I*) was maintained at 0.15 M using sodium chloride (NaCl) as a background electrolyte. All experiments were performed at least in triplicate to ensure reproducibility.

#### 3.3.2 Kinetics Experiments

The formation kinetics of the Ga^3+^-HBCD complexes was evaluated at room temperature using ^1^H NMR and UV–vis spectroscopies. Equimolar amounts of Ga^3+^ and HBCD solutions (final concentrations: 1 mM for ^1^H NMR, 50 μM for UV–vis) in buffered media (e.g., pH 2 HCl 10^–2^ M, pH 4.5 acetic/acetate buffer). The complexation reaction was tracked by monitoring the increase in peaks characteristic of Ga^3+^-ligand complex formation over time.

#### 3.3.3 Thermodynamic Experiments

UV–vis pH-spectrophotometric titrations were conducted using the in-cell method for the free HBCD and the out-of-cell method for the Ga^3+^-HBCD mixture at *T* = 25 °C and *I* = 0.15 M NaCl. In the latter case, stock solutions of HBCD and Ga^3+^ were combined in separate vials at 1:1 metal-to-ligand ratio (*C*_Ga_ = *C*_L_ = 20–50 μM). Small volumes (μL) of HCl and/or NaOH were used to adjust the pH. pH measurements were performed using a calibrated pH meter (Mettler-Toledo). The vials were sealed and heated to *T* = 80 °C to ensure complete complexation, then cooled to ambient temperature. The electronic spectra were recorded, and the equilibrium was considered to be reached when no further changes in either the pH or the electronic spectra were observed.

^1^H NMR spectra of free HBCD or Ga^3+^-HBCD mixtures (*C*_L_ = *C*_Ga_ = 1 mM) were recorded at *T* = 25 °C and *I* = 0.15 M NaCl, in D_2_O, at different pH. The pH was adjusted following the procedure used in the UV–vis experiments. To account for isotopic effects, 0.41 log units were added to the measured pH values. Under highly acidic conditions, the pH was calculated from the HCl concentration using the formula pH = −log­[*C*(H^+^)]. The equilibrium for metal complexation was considered to be reached when no further changes were observed in either the ^1^H NMR spectra or the pH readings. Prolonged heating was applied to accelerate the complexation reaction. The thermodynamic data were elaborated with HypSpec, HypNMR or as described in previous publications.^45,48,49^ Hydrolysis constants and solubility products of Ga^3+^ in aqueous ionic media were taken from ref 49.

### 3.4 DFT

DFT calculations were performed using B3LYP/6-31+G­(d,p), as implemented in Gaussian09, with SCRF implicit hydration.^50–52^ ^1^H NMR and ^13^C NMR spectra were simulated using DFT following the procedure outlined the literature.^53,54^ Specifically, geometries were optimized at the B3LYP/6-31 + G­(d,p) level, and nuclear magnetic shielding were computed at the GIAO mPW1PW91/6-311 + G­(2d,p) level. As per the literature procedure, geometries were optimized in the gas phase, while NMR response was computed with implicit solvation. The effect of implicit solvation on geometries was also tested; however, for the NMR spectra, regression parameters reported for gas-phase optimizations were used. The reported root-mean-square deviation (RMSD) for these estimates is 0.16 ppm for ^1^H and 2.49 ppm for ^13^C.^53,54^ The reported DFT spectra do not include *J*–*J* coupling, hence computed ^1^H signals appear as single peaks.

To check the importance of the selected DFT method, the most stable geometries were optimized also at the TPSSTPSS/6-31 + G­(d,p)^46,55,56^ level including Grimme D3 empirical dispersion^47^ and implicit solvation based on the SAS molecular surface, as implemented in Gaussian.

### 3.5 Gallium-68 Radiolabeling

A 1850 MBq ^68^Ge/^68^Ga generator (GalliaPharm, Ezag, Berlin) was manually eluted with 0.1 M HCl (5 mL). HBCD and HBED stock solutions were prepared in ultrapure water at 1.0 × 10^–3^ M and diluted appropriately to give a serial dilution series (1.0 × 10^–4^ to 1.0 × 10^–8^ M).

Radiolabeling experiments were performed by adding the generator-produced [^68^Ga]­Ga^3+^ (1–10 MBq, 10 μL) to a solution containing the ligand (10 μL) at the appropriate concentration, along with a proper buffering salt depending on the desired final pH (pH 3.5:21 μL sodium acetate 0.1 M; pH 4.5:15 μL sodium acetate 0.1 M; pH 7:30 μL NaH_2_PO_4_ 0.1 M). The final reaction volume was adjusted to 100 μL with ultrapure water. Different apparent molar activities were tested from 0.1 to 10,000 MBq/nmol, corresponding to a final ligand concentration ranging from 1.0 × 10^–4^ to 1.0 × 10^–9^ M. Reactions were conducted at ambient temperature or 90 °C for 5 or 15 min. All radiolabeling reactions were repeated at least three times.

Radiochemical incorporation (RCI) was determined via radio-thin layer chromatography (radio-TLC) on silica (silica gel 60 F_254_ aluminum plates), using methanol/NH_4_OAc 1 M 1/1 *V*/*V* as the eluent. Under these conditions, [^68^Ga]­Ga^3+^-complexes migrates with the solvent front (*R*_f_ = 0.5–0.6) while free [^68^Ga]­Ga^3+^ remains at the baseline (*R*_f_ = 0). A Cyclone Plus Storage Phosphor System (PerkinElmer) was employed to analyze the radio-TLC plates after exposure to a super-resolution phosphor screen (type MS, PerkinElmer; Waltham, MA, USA). Data were processed with OptiQuant software (version 5.0, PerkinElmer Inc.; Waltham, MA, USA).

### 3.6 Stability under Biological Conditions

The stability of the preformed complexes ([^68^Ga]­[Ga­(HBCD)]^−^ and [^68^Ga]­[Ga­(HBED)]^−^) was evaluated over time by incubating them in PBS or human serum (1:1 *V*/*V* dilution) at *T* = 37 °C. The radiometal-complex stability was monitored at various time points over 2 h using radio-TLC, following the protocol described earlier.

## 4 Conclusions

In this study, we investigated the HBCD chelator, a structurally modified derivative of HBED, in which a rigidity element has been introduced. This structural modification effectively prevents the formation of multiple isomers upon Ga^3+^ complexation, a critical improvement over HBED. Preventing isomerization is essential to ensure that the resulting [^68^Ga]­Ga-labeled bioconjugates maintain consistent biological profiles.

Our results confirmed that incorporating the cyclohexane diamine scaffold leads to the formation of a single-isomeric hexacoordinated (*cis*–*trans* N_2_O_4_) Ga^3+^ complex. Although Ga^3+^-HBCD is less thermodynamically stable than HBED, it remains more stable than complexes formed with other clinically employed ^68^Ga-chelators, such as DOTA.

Moreover, HBCD demonstrated the ability to bind [^68^Ga]­Ga^3+^ under highly diluted radiochemical conditions. While HBED slightly outreaches the labeling performance of HBCD—likely due to steric hindrance from the DACH scaffold—[^68^Ga]­[Ga­(HBCD)]^−^ exhibits equivalent remarkable stability than [^68^Ga]­[Ga­(HBED)]^−^ in biological media, including human serum.

These findings underscore the potential of HBCD as a promising chelator for the development of innovative ^68^Ga-labeled PET radiotracers. By effectively overcoming the isomerism issue associated with HBED, HBCD paves the way for more reliable and consistent radiotracer development, ultimately advancing the field of molecular imaging.

## Supplementary Material



## Notes

NMR data (NMR spectra of intermediates and HBCD in organic solvents, mono and bidimensional NMR spectra of HBED and Ga^3+^-HBCD aqueous solutions at different conditions—pH, time points etc.), speciation diagram of HBCD and HBED, UV–vis data (spectra of Ga^3+^-HBCD at different pH), MS data (spectra of HBCD and Ga^3+^-HBCD) and DFT data (DFT-optimized structure of Ga^3+^-HBCD isomers and calculated NMR spectra) (PDF)
