# Supplementary material for: Thwarting Isomerization through Rigidity: A Promising HBED Derivative for the Chelation of Gallium-68
Source: Inorg Chem. 2025 Jul 17;64(37):18673–86. doi: 10.1021/acs.inorgchem.5c00930 (PMC12458685; doi:10.1021/acs.inorgchem.5c00930)
Supplement: Supplementary file 1 [file ic5c00930_si_001.pdf]

Supporting Information

Thwarting Isomerization through Rigidity: A Promising HBED Derivative for the Chelation of Gallium-68

Marianna Tosato[1], Matteo Boniburini[2], Francesco Faglioni[2], Francesco Genua[2], Matteo Mari[2], Jennifer Storchi[2], Sara Franchi[3], Mattia Asti[1] and Erika Ferrari[2,*]

[1] Radiopharmaceutical Chemistry Laboratory, Nuclear Medicine Unit, AUSL-IRCCS Reggio Emilia, 42122 Reggio Emilia (Italy)

[2] Department of Chemical and Geological Sciences, University of Modena and Reggio Emilia, 41125 Modena (Italy)

[3] Department of Chemical Sciences, University of Padova, 35131 Padova (Italy)

*** Corresponding author**: erika.ferrari@unimore.it

Supporting Figures

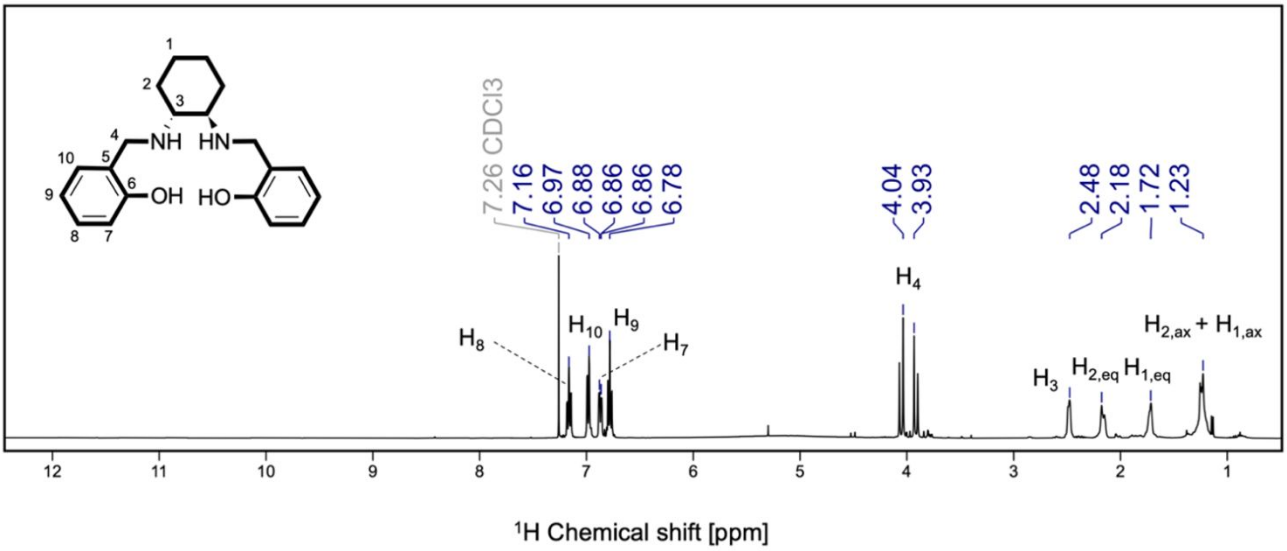

Figure S1. ^{1}H NMR spectrum of N,N′-di(2-hydroxybenzyl)-1,2-cyclohexanediamine (400 MHz, $CDCl_3$, T = 25°C) and signal attributions. *Note: Signal assignments for all compounds were determined using bidimensional spectra (^{1}H-^{1}H COSY, ^{1}H-^{13}C HSQC, and ^{1}H-^{13}C HMBC), with the data omitted for brevity.*

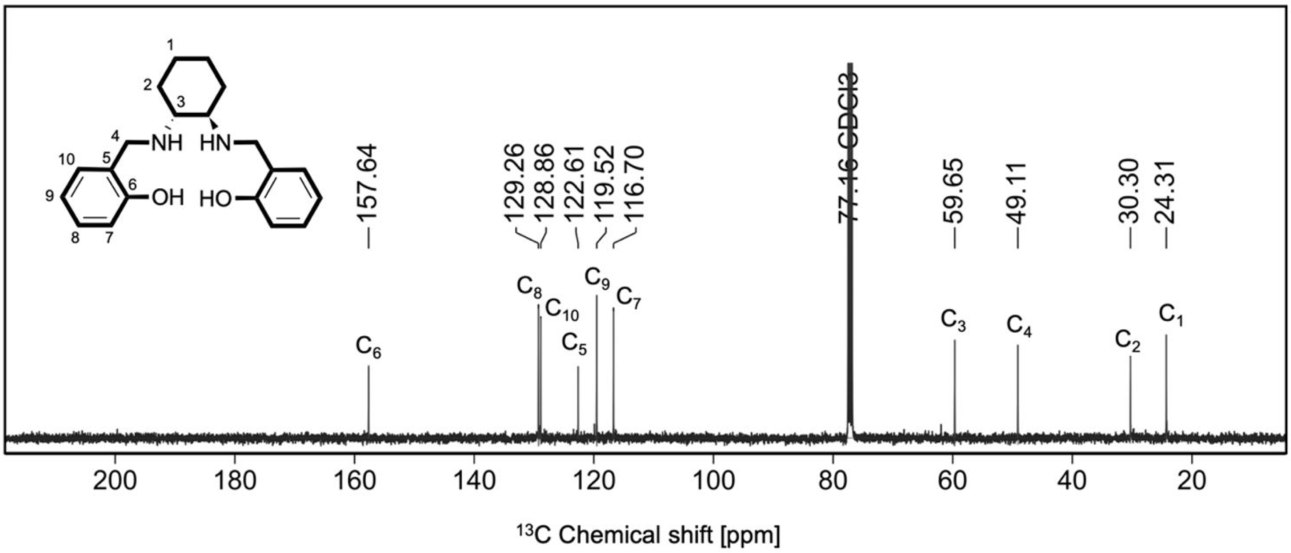

Figure S2. ^{13}C{^{1}H} NMR spectrum of N,N′-di(2-hydroxybenzyl)-1,2-cyclohexanediamine (400 MHz, $CDCl_3$, T = 25°C) and signal attributions.

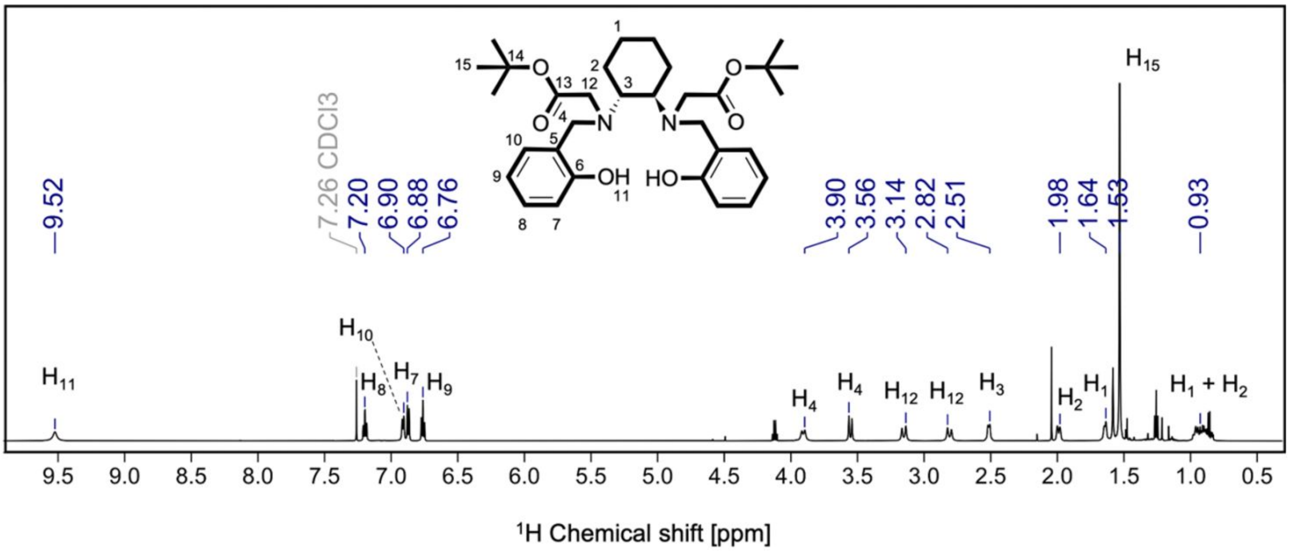

Figure S3. ^{1}H NMR spectrum of N,N'-di(2-hydroxybenzyl)-(1,2-cyclohexanediamine)-N,N-diacetic acid di-*t*-butyl ester (600 MHz, $CDCl_3$, *T* = 25°C) and signal attributions.

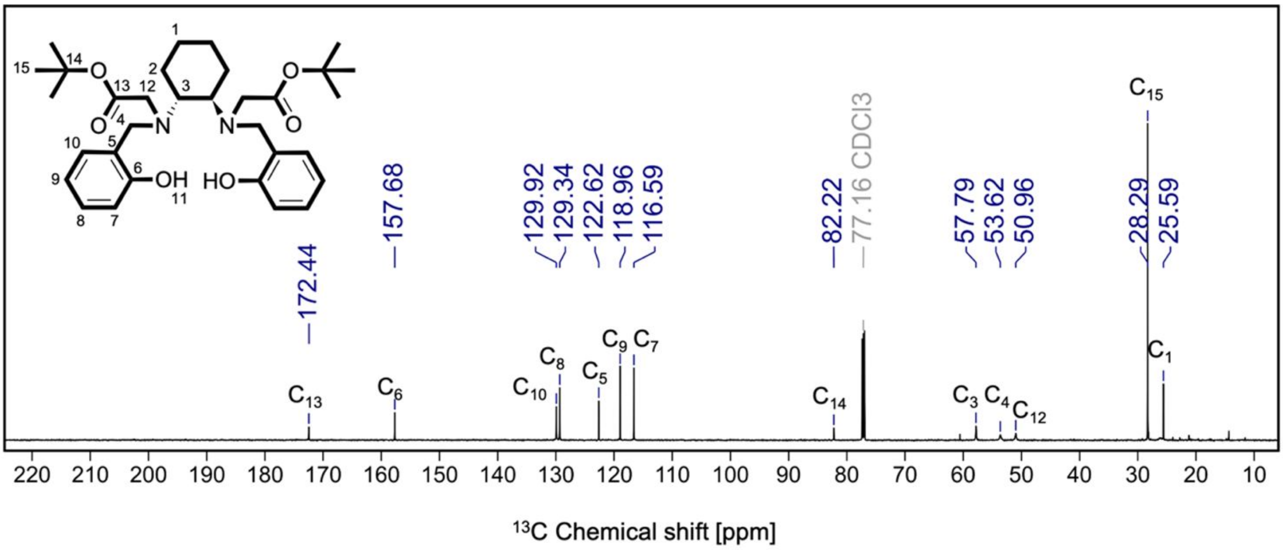

Figure S4. $^{13}C\{^{1}H\}$ NMR spectrum of N,N'-di(2-hydroxybenzyl)-(1,2-cyclohexanediamine)-N,N-diacetic acid di-*t*-butyl ester (600 MHz, $CDCl_3$, *T* = 25°C) and signal attributions.

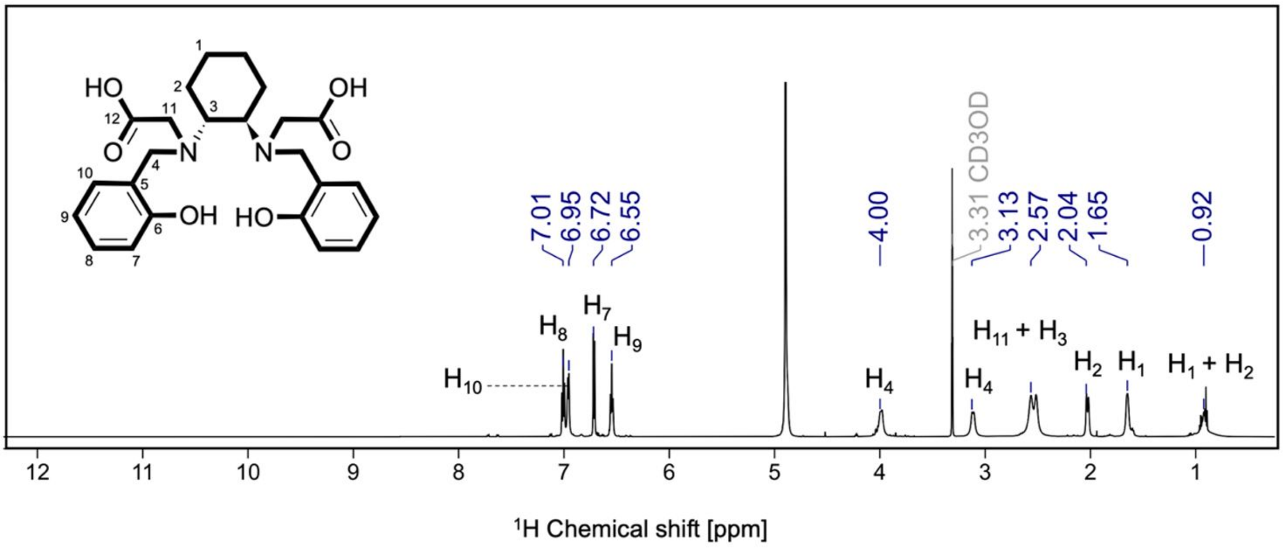

Figure S5. ^{1}H NMR spectrum of HBCD (600 MHz, CD_3OD, T = 25°C) and signal attributions.

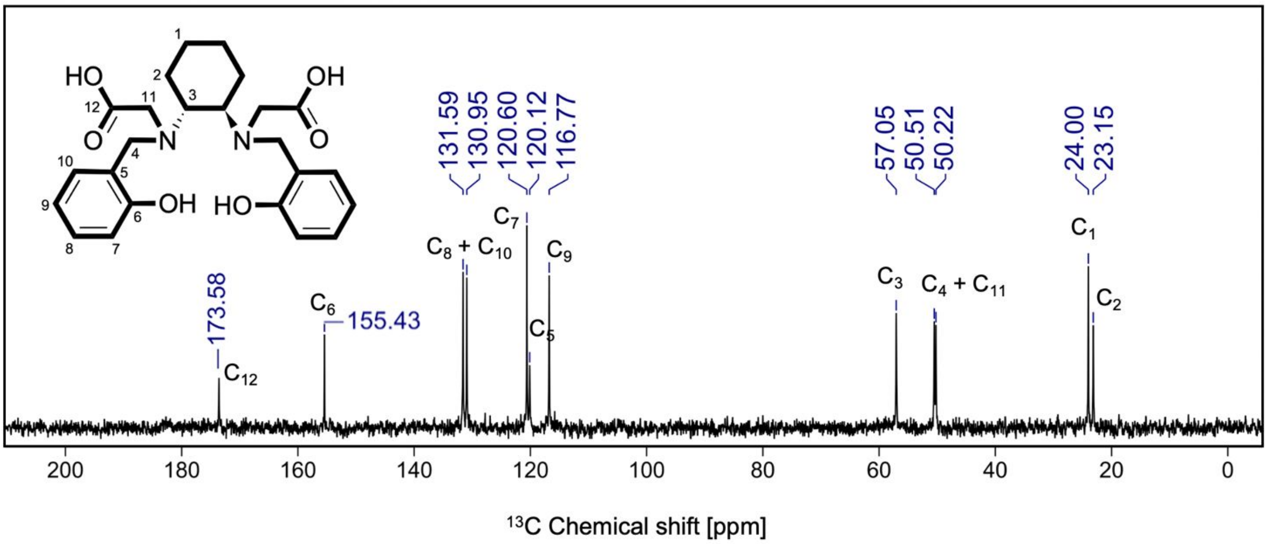

Figure S6. $^{13}C\{^{1}H\}$ NMR spectrum of HBCD (600 MHz, D_2O, pH 6.7, T = 25°C) and signal attributions.

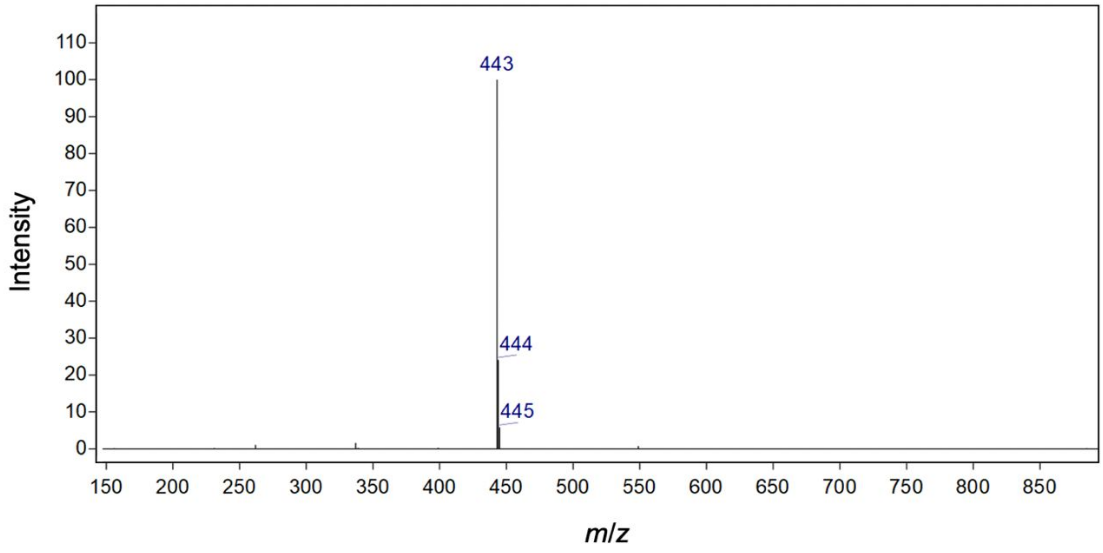

Figure S7. ESI-MS spectrum of HBCD.

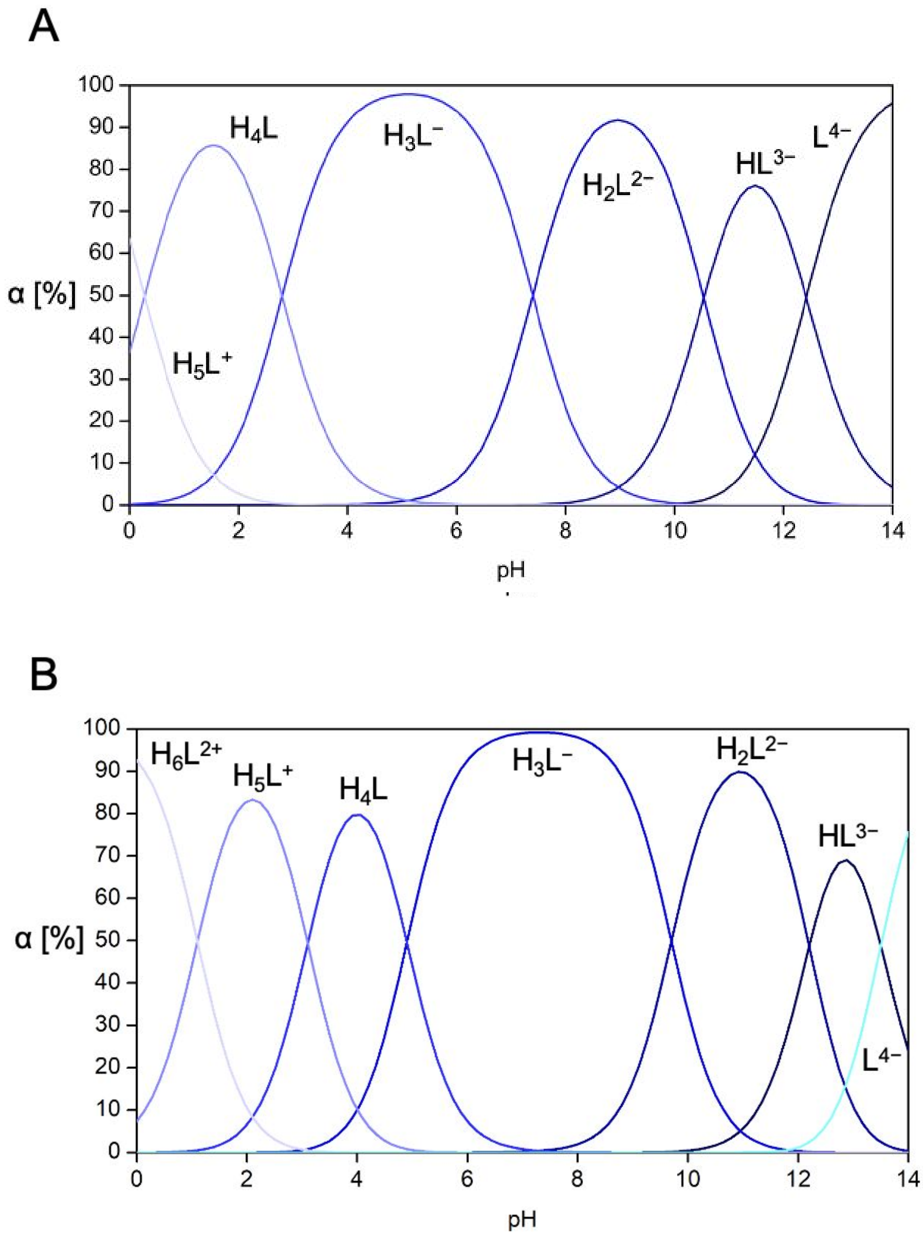

Figure S8. Distribution diagram of (A) HBED and (B) HBCD. For HBCD, the species distribution diagram was predicted using protonation constants calculated from ^{1}H NMR data (Table 1).

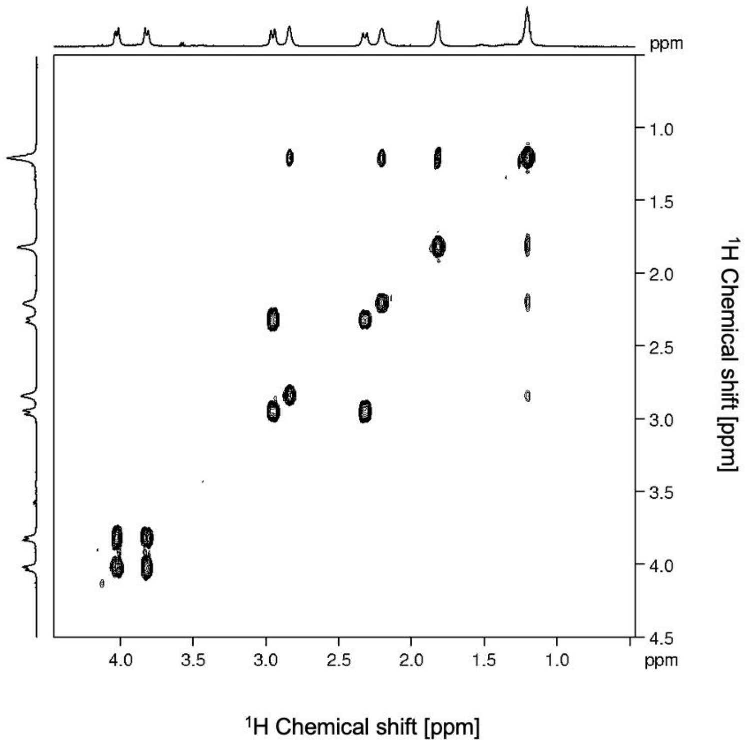

Figure S9. ^{1}H-^{1}H COSY spectrum - aliphatic region (400 MHz, D_2O, T = 25°C, I = 0.15 M NaCl, pH = 6.33) of HBCD.

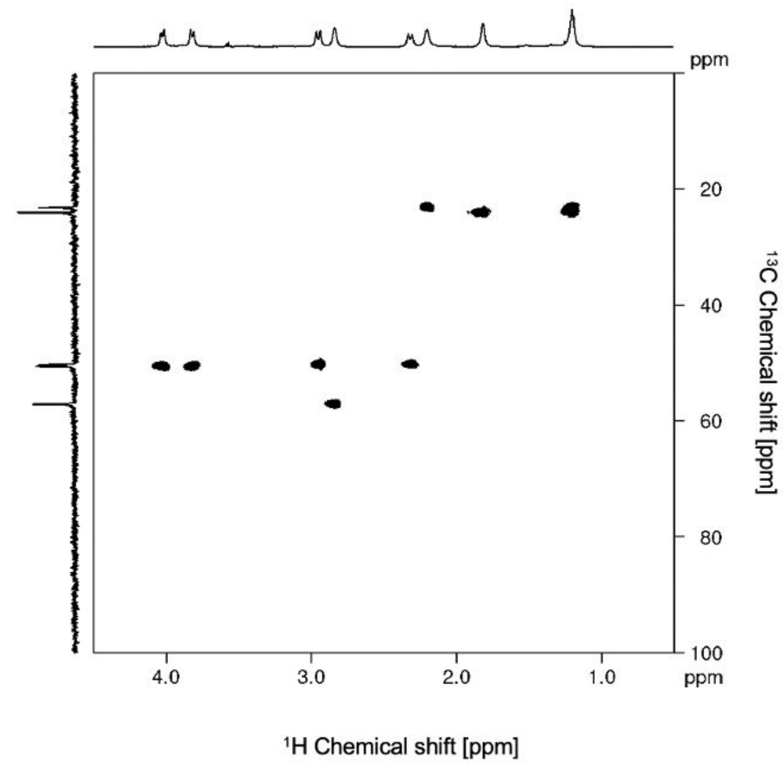

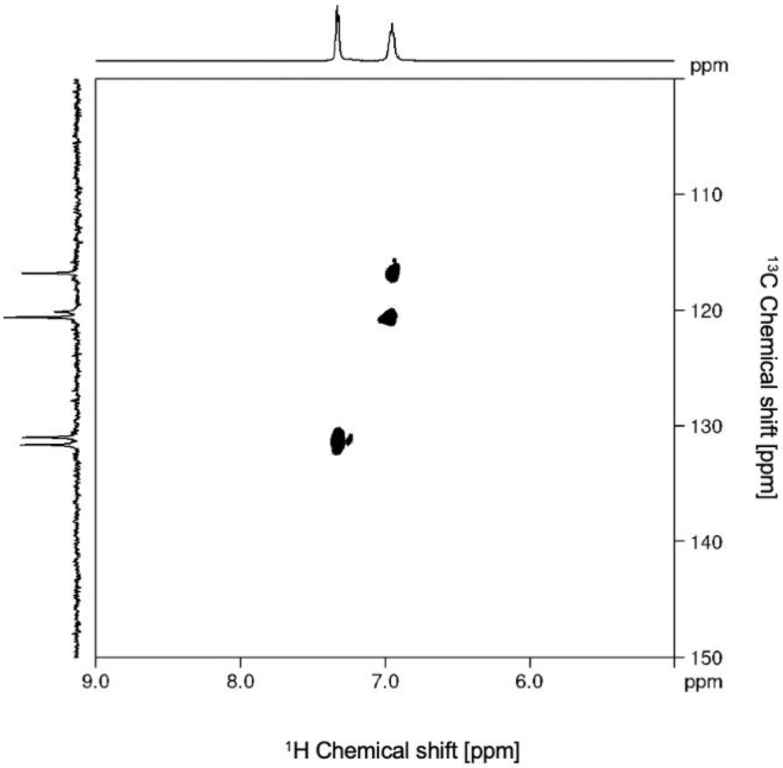

Figure S10. ^{1}H-^{13}C HSQC spectrum (400 MHz, D_2O, T = 25°C, I = 0.15 M NaCl, pH = 6.33) of HBCD.

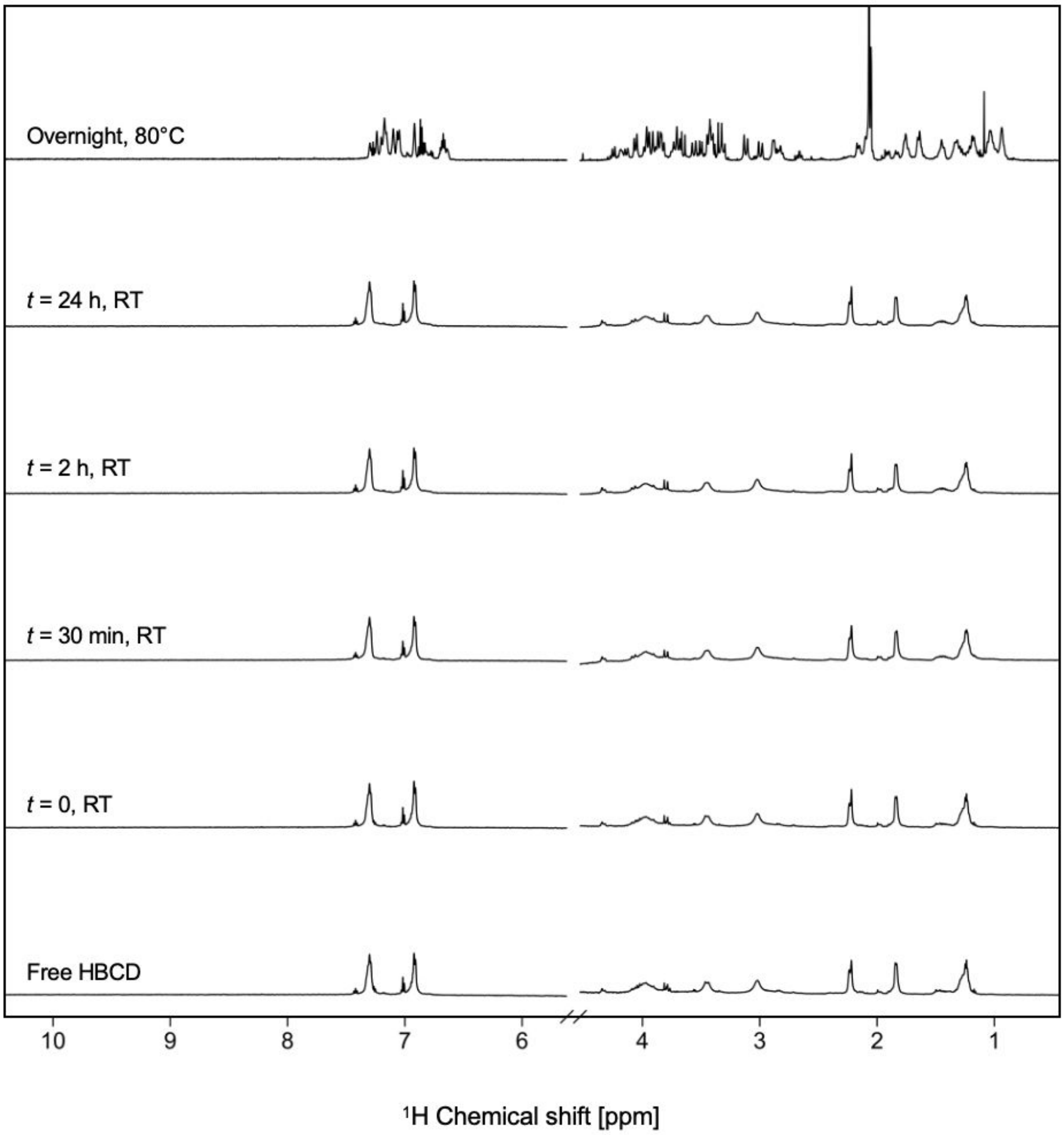

Figure S11. Time- and temperature-dependent ^{1}H NMR spectra (600 MHz, D_2O, T = 25°C) of Ga^{3+}-HBCD mixture (C_{Ga} = C_L = 1 mM) at pH ~ 2.

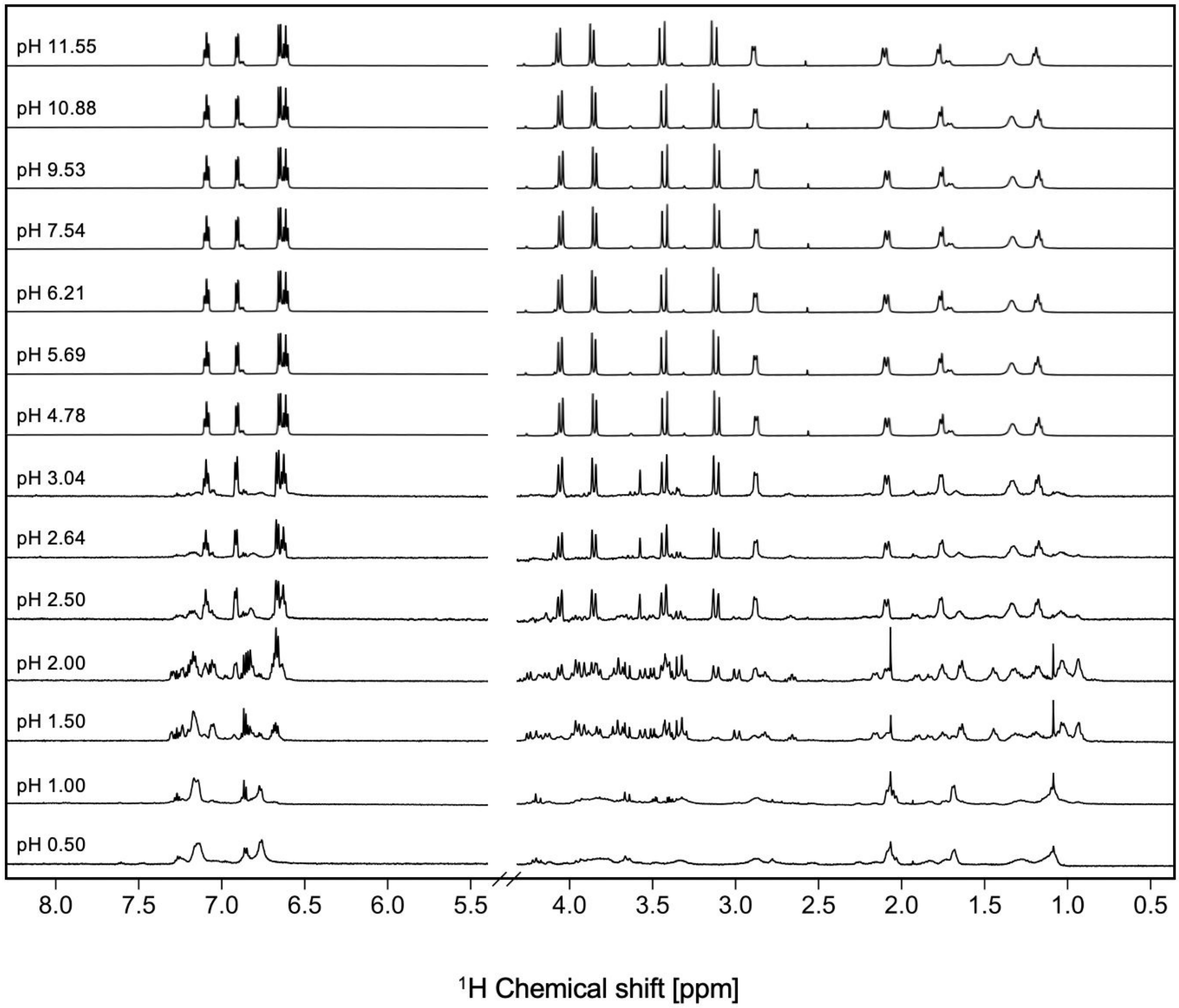

Figure S12. Representative variable-pH ^{1}H NMR spectra of Ga^{3+}-HBCD (600 MHz, D_2O, T = 25°C, C_{Ga} = C_L = 1 mM, I = 0.1 M NaCl).

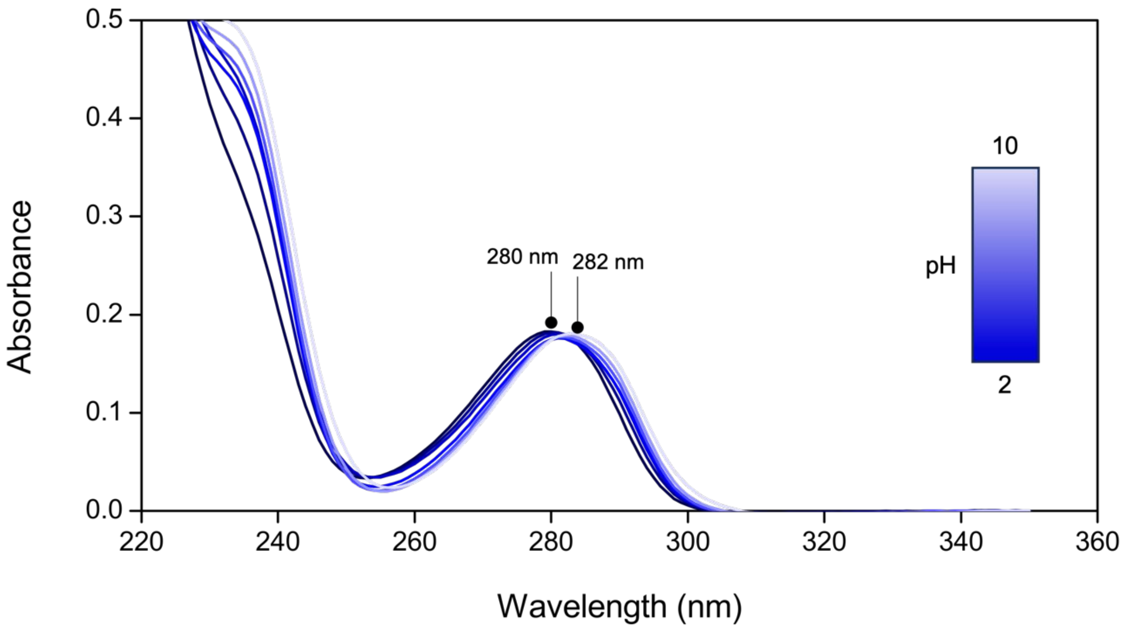

Figure S13. Representative variable-pH UV-Vis spectra of Ga^{3+}-HBCD (T = 25°C, C_{Ga} = C_L = 16 mM, I = 0.1 M NaCl).

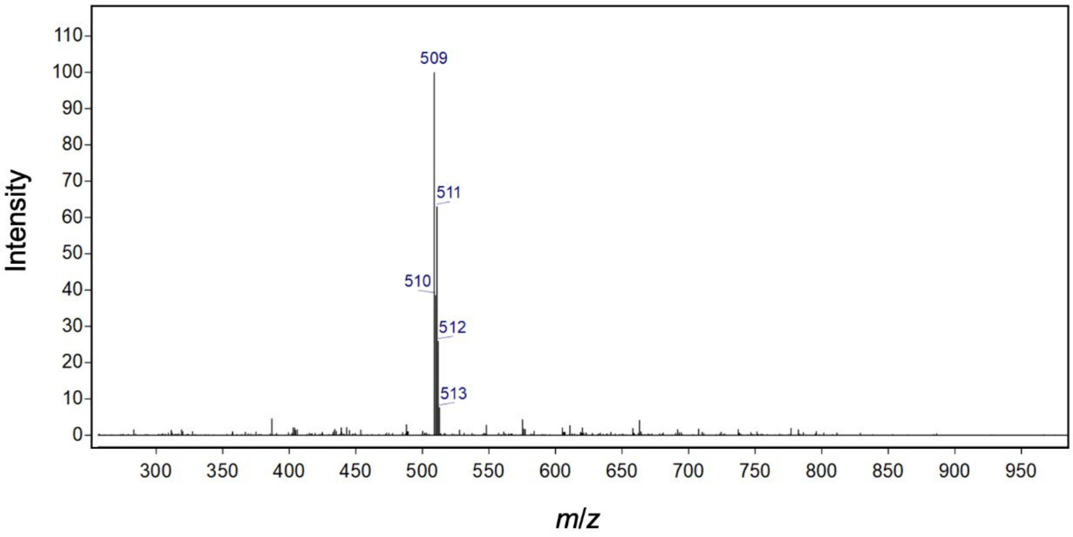

Figure S14. ESI-MS spectrum of Ga^{3+}-HBCD.

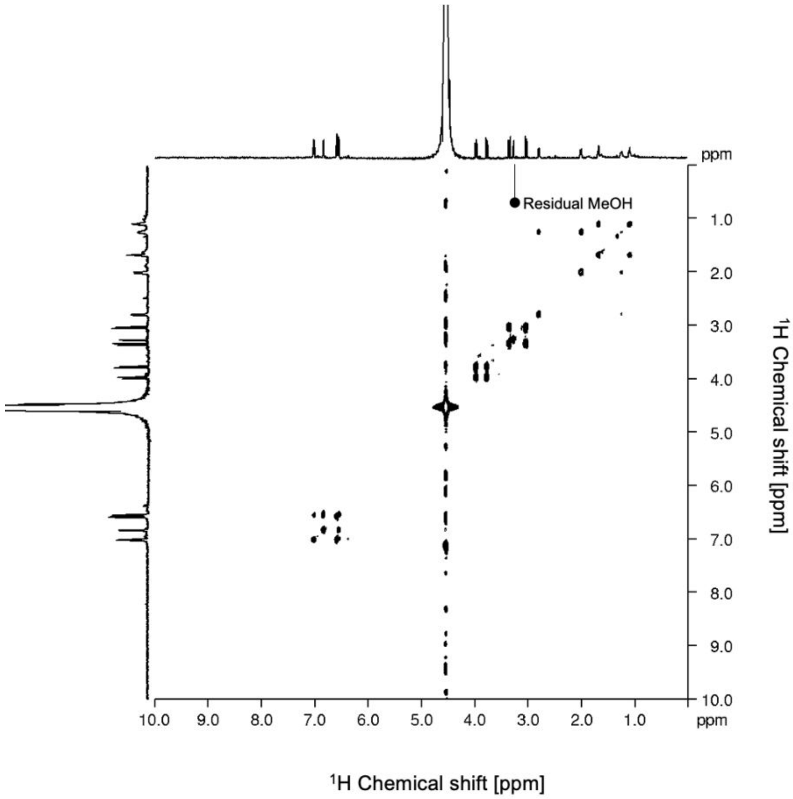

Figure S15. ^{1}H-^{1}H COSY spectrum of $[GaL]^-$ (L = HBCD, 600 MHz, D_2O, *T* = 25°C).

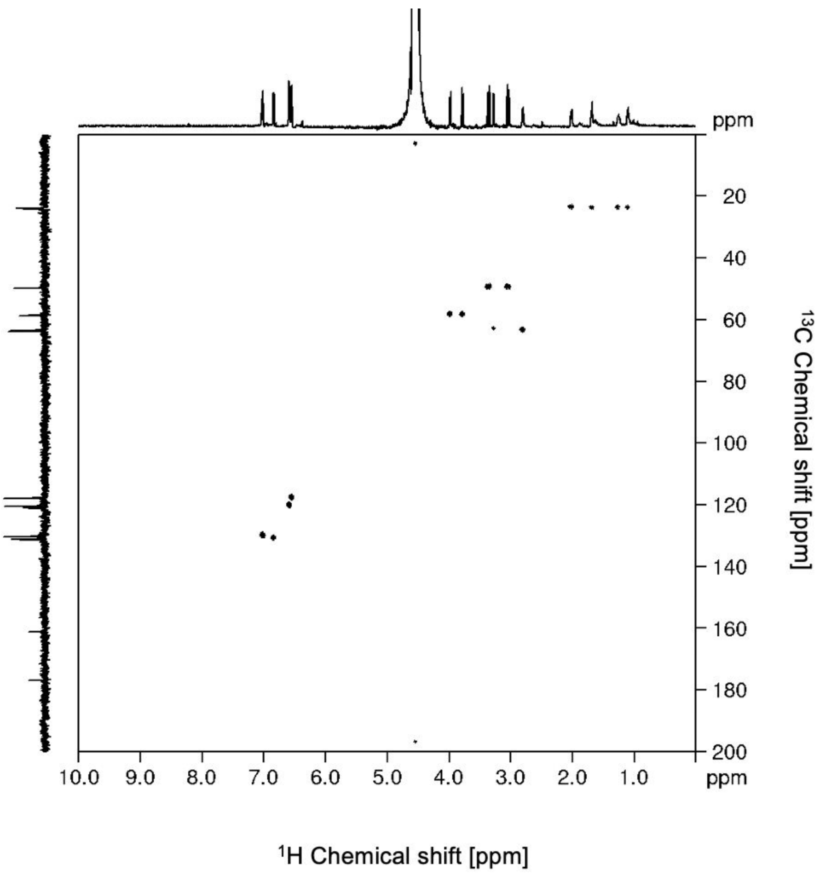

Figure S16. ^{1}H-^{13}C HMQC spectrum of $[GaL]^{-}$ (L = HBCD, 600 MHz, D_2O, T = 25°C).

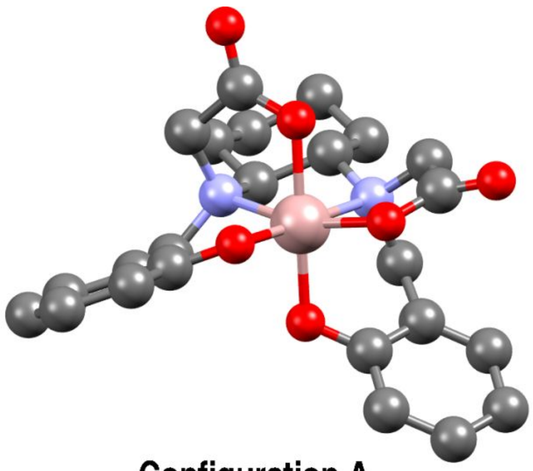

Configuration A

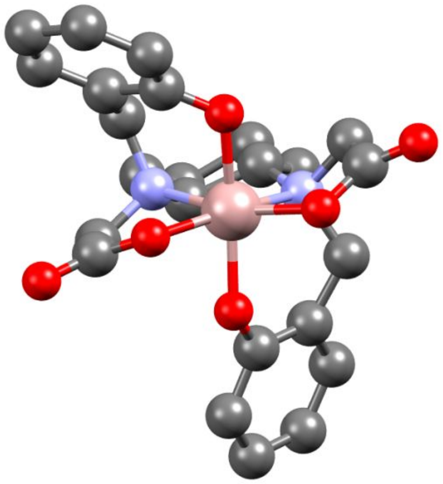

Configuration C

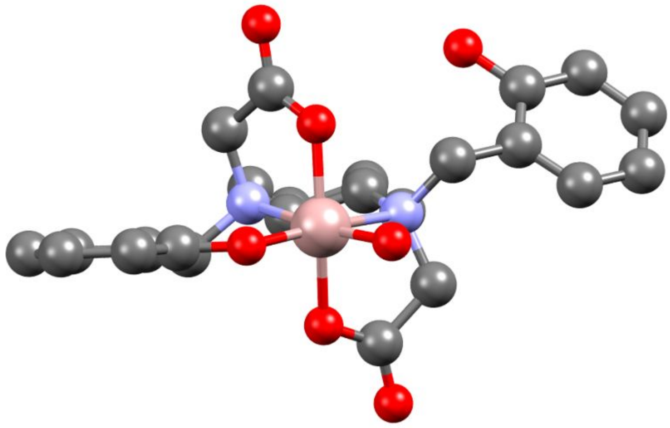

Configuration D

Figure S17. DFT-optimized structures of $[GaL]^-$ (configuration A, C and D).

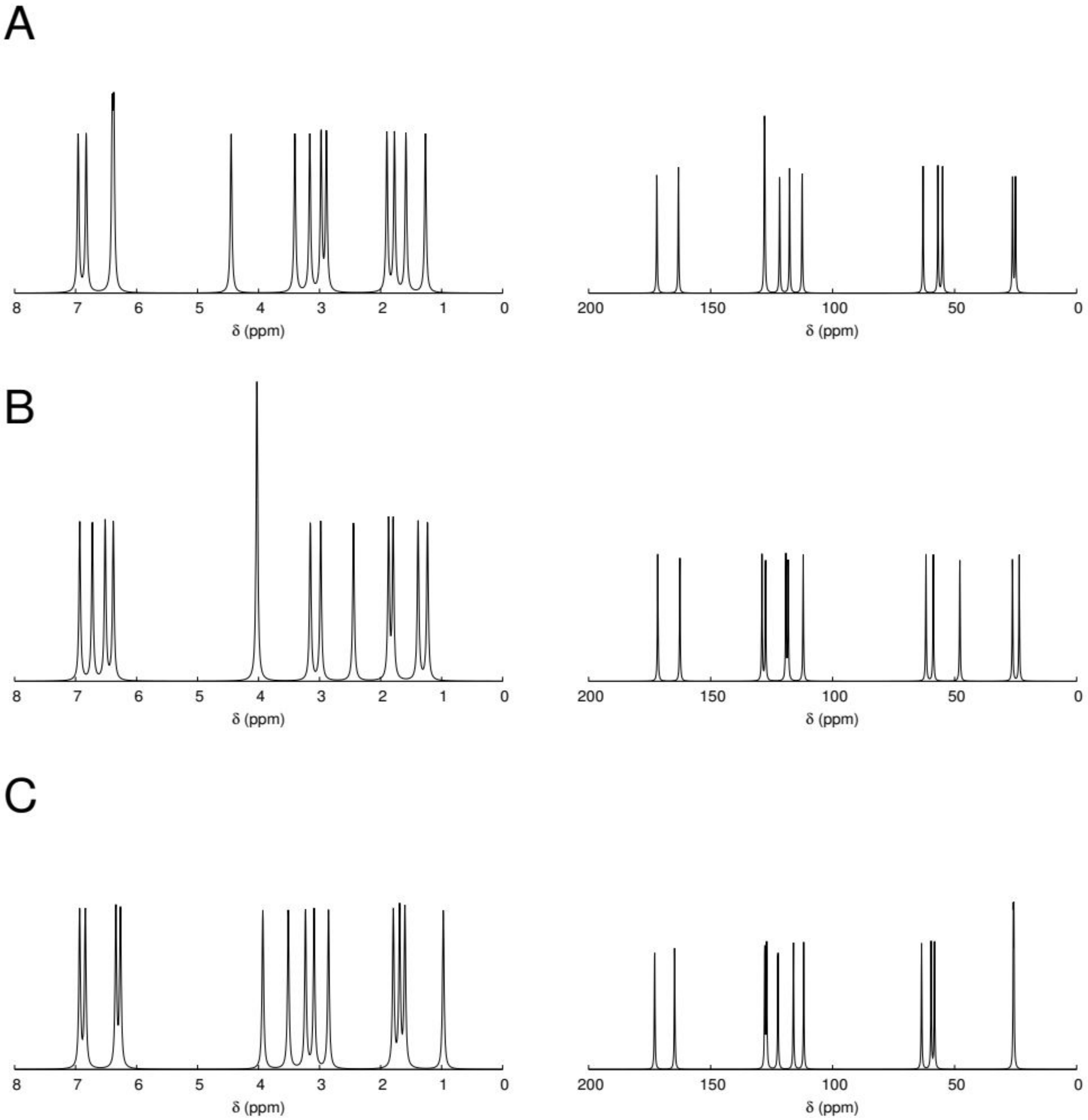

Figure S18. Simulated ^{1}H (left) and ^{13}C (right) NMR spectra of 6-coordinated $[GaL]^{-}$ (L = HBCD): (A) isomer A, (B) isomer B and (C) isomer C. Full width at half maximum was set arbitrarily to 0.3 ppm to enhance visualization.

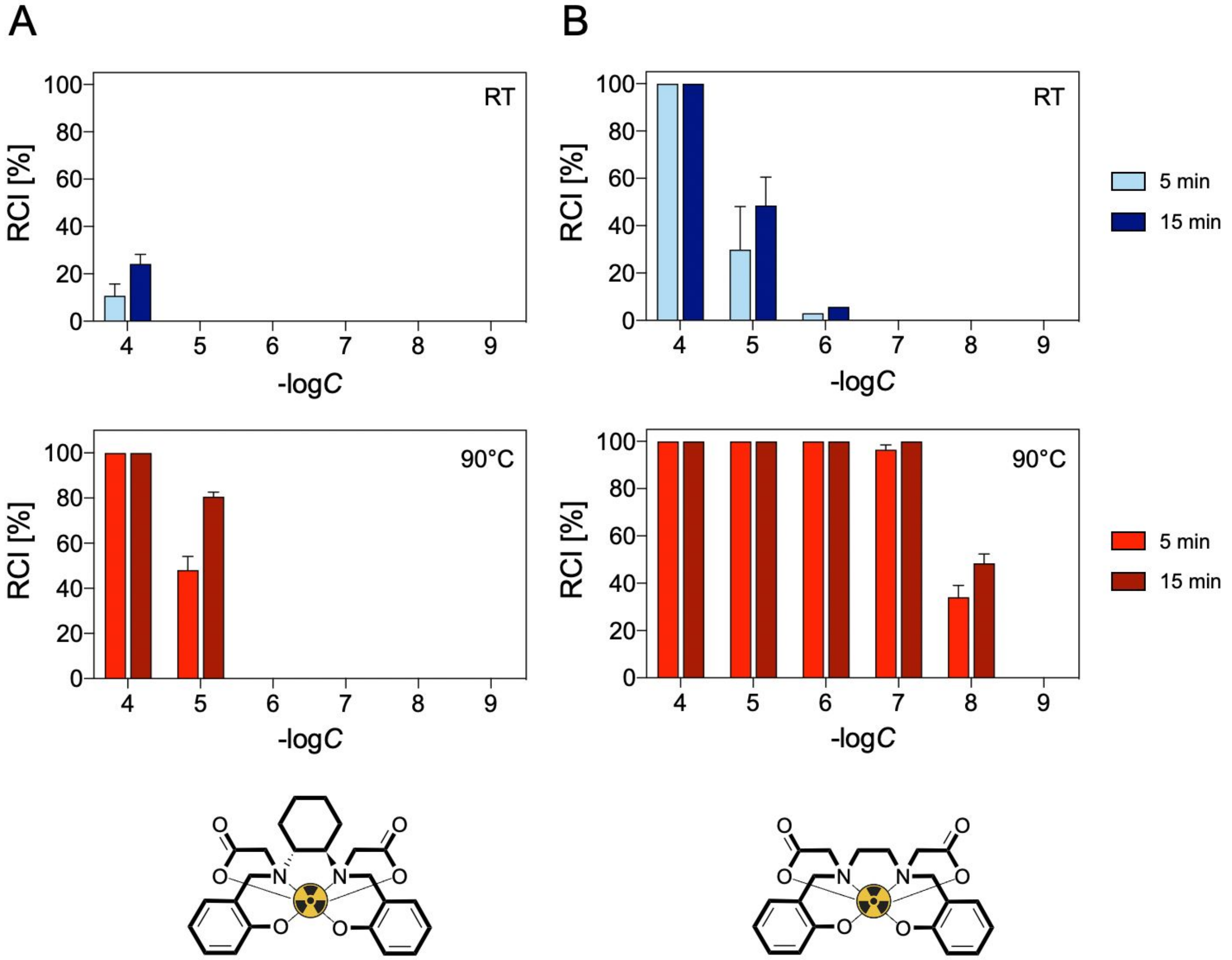

Figure S19. Concentration-, time- and temperature-dependent [^{68}Ga]Ga^{3+} RCIs of (A) HBCD and (b) HBED at pH 3.

Supporting Tables

Table S1. DFT-calculated ^{1}H chemical shifts for 6-coordinated $[GaL]^{-}$ (L = HBCD) isomers.

Proton	Chemical shift [ppm]		
	Isomer A	Isomer B	Isomer C
1_a	1.265	1.231	0.972
1_e	1.772	1.794	1.601
2_a	1.584	1.385	1.688
2_e	1.897	1.868	1.791
3	2.888	2.444	2.854
$4_{\_1}$	3.405	4.027	3.090
$4_{\_2}$	4.450	4.027	3.930
7	6.399	6.515	6.265
8	6.958	6.932	6.934
9	6.373	6.382	6.340
10	6.826	6.725	6.841
$11_{\_1}$	2.975	2.982	3.231
$11_{\_2}$	3.160	3.152	3.513

(a) and (e) indicate axial and equatorial positions.

Table S2. DFT-calculated ^{13}C chemical shifts for 6-coordinated $[GaL]^{-}$ (L = HBCD) isomers.

Carbon	Chemical shift [ppm]		
	Isomer A	Isomer B	Isomer C
1	26.264	26.281	25.927
2	25.061	23.525	25.696
3	62.939	61.739	63.547
4	56.823	58.740	59.623
5	121.713	119.179	122.407
6	163.197	162.608	164.749
7	117.637	118.218	116.068
8	127.781	127.501	127.804
9	112.495	112.068	111.859
10	127.980	128.951	127.036
11	54.942	47.858	58.239
12	172.044	171.686	172.932

Table S3. TPSS-D3-calculated relative energies for possible $[GaL]^-$ configurations.

	Configuration		Relative Energy (kcal/mol)
6 donors	A	PhO *cis,* COO *cis*	4.8
	B	PhO *cis,* COO *trans*	0.0
	C	PhO *trans,* COO *cis*	11.3
5 donors + H_2O	D	COO *trans*	11.6
